# Supplementary material for: Vaccine-Induced Protection from Homologous Tier 2 SHIV Challenge in Nonhuman Primates Depends on Serum-Neutralizing Antibody Titers
Source: Immunity. 2019 Jan 15;50(1):241–252.e6. doi: 10.1016/j.immuni.2018.11.011 (PMC6335502; doi:10.1016/j.immuni.2018.11.011)
Supplement: Document S1. Figures S1–S7 and Tables S1–S3 [file mmc1.pdf]

Immunity, Volume *50*

Supplemental Information

Vaccine-Induced Protection from Homologous Tier 2 SHIV Challenge in Nonhuman Primates Depends on Serum-Neutralizing Antibody Titers

Matthias G. Pauthner, Joseph P. Nkolola, Colin Havenar-Daughton, Ben Murrell, Samantha M. Reiss, Raiza Bastidas, Jérémie Prévost, Rebecca Nedellec, Benjamin von Bredow, Peter Abbink, Christopher A. Cottrell, Daniel W. Kulp, Talar Tokatlian, Bartek Nogal, Matteo Bianchi, Hui Li, Jeong Hyun Lee, Salvatore T. Butera, David T. Evans, Lars Hangartner, Andrés Finzi, Ian A. Wilson, Richard T. Wyatt, Darrell J. Irvine, William R. Schief, Andrew B. Ward, Rogier W. Sanders, Shane Crotty, George M. Shaw, Dan H. Barouch, and Dennis R. Burton

SUPPLEMENTARY MATERIALS

Figure S1: Selection of challenge animals from previously immunized macaques.
Figure S2: Viral load of unimmunized animals after viral challenge.
Figure S3: NAb titers from protected animals remain stable over one year past immunization
Figure S4: Neutralizing antibody titers 7 days before the detection of viremia.
Figure S5: Bayesian logistical regression of serum nAb titer and infection probablility.
Figure S6: Additional analyses of $CD4^+$ and $CD8^+$ T cell activation at week 0.
Figure S7: Additional analysis of ADCC activity from control mAbs and animal sera at week 0.

Table S1: Genotyping of macaques used in the immunization study.
Table S2: Estimated frequency of MOI in macaques following SHIV challenge.
Table S3: Survival models and statistical end point analyses.

A

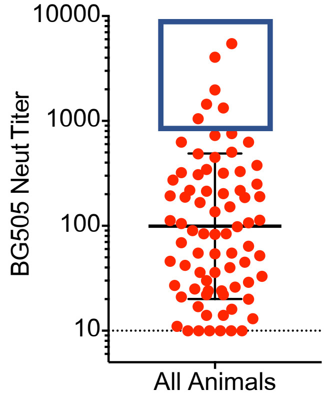

B

Immunogen	Animal ID	Peak BG505 nAb-titer		Animal ID	Immunogen
BG505 SOSIP v4.1	12-137	1979	54	12-060	BG505 SOSIP v4.1
BG505 SOSIP v4.1	12-046	1328	90	12-065	BG505 SOSIP v5.2
BG505 Olio6	12-143	1048	40	12-149	BG505 Olio6 CD4-KO
BG505 SOSIP v5.2 (Pump)	12M169	4047	25	12-153	BG505 SOSIP v5.2
BG505 SOSIP v5.2 (Pump)	11M088	1440	64	12M248	BG505 SOSIP v5.2 (Pump)
BG505 Olio6 CD4-KO (Lipo)	4O9	5453	13	0Q7	BG505 SOSIP.664 His (Lipo)

C

	Animal ID	Peak Titer nAb-titer	Gender	Age [y]	Weight [kg]
High nAb-Titer Group	12-137	1979	Female	4.3	4.1
	12-046	1328	Male	4.5	5.3
	12-143	1048	Female	4.1	3.3
	12M169	4047	Female	4.4	4.2
	11M088	1440	Female	5.5	7.4
	4O9	5453	Female	4.0	4.5
Low nAb-Titer Group	12-060	54	Male	4.4	5.7
	12-065	90	Female	4.5	4.5
	12-149	40	Female	4.0	3.6
	12-153	25	Female	4.0	3.8
	12M248	64	Female	4.4	4.7
	0Q7	13	Female	4.1	5.0
			**Average**	**4.4**	**4.7**
			**SD**	**0.4**	**1.1**

Figure S1, related to Figure 1. Selection of challenge animals from previously immunized macaques. (A-C) Challenge animals were selected from 78 macaques previously immunized with the indicated native-like BG505 trimer proteins (de Taeye et al., 2015; Kulp et al., 2017; Sanders et al., 2013; Torrents de la Peña et al., 2017) at weeks 0, 8 and 24 (Pauthner et al., 2017). Immunogens used in the study differ only in the precise details of the modifications made to stabilize the V3 loop; detailed accounts of differences can be found in the respective publications cited above. Some trimers additionally included a mutation to reduce binding to human CD4 (CD4-KO), which

minimally affects rhesus CD4 binding. All bolus-immunized animals received 100μg adjuvanted trimer per immunization; osmotic pump-immunized animals (Pump) received 200μg adjuvanted trimer per immunization. (A) 6 macaques with the highest peak autologous BG505 neutralization titers at week 26 were selected for the high nAb titer group in this study as shown in the blue box. (**B**) High nAb titer animals were carefully matched with animals that received identical or very similar immunization regimens but had developed low peak (week 26) autologous nAb titers. (**C**) High- and low-titer animals were closely matched for gender, age and weight.

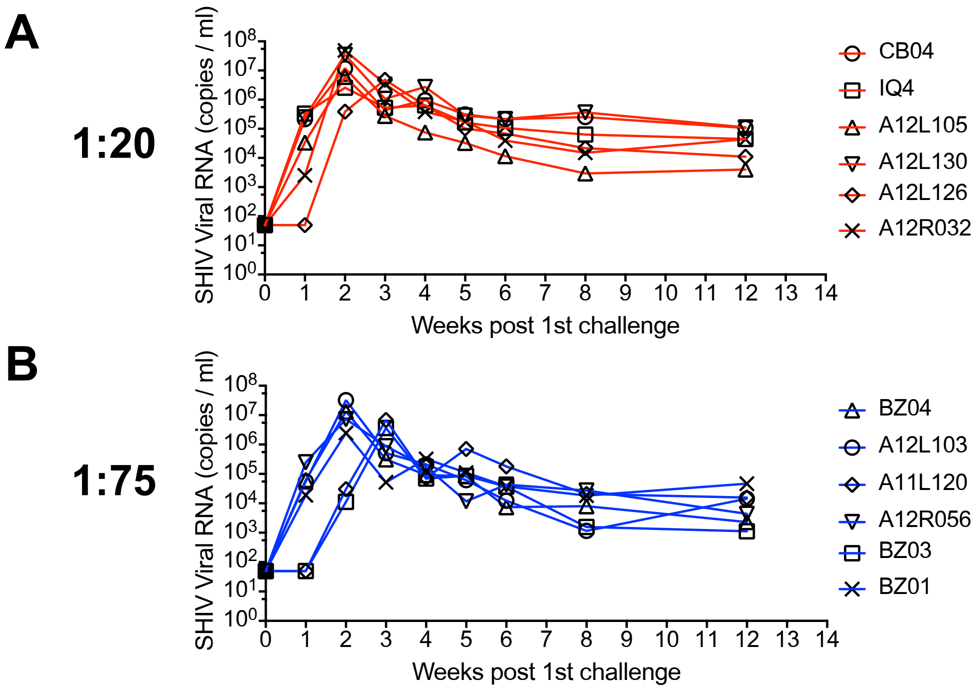

Figure S2, related to Figure 2. Viral load of unimmunized animals after viral challenge. Viral load curves of the 1:20 (1 x 10^8 RNA copies/ml) (**A**) and 1: 75 (2.8 x 10^7 RNA copies/ml) titration control group animals (**B**). 2.8 x 10^7 viral RNA copies/ml, or 1.4 x 10^7 virions, infected at least 4/6 animals with the 1st challenge and the remaining 2 animals following the 2nd challenge. The 1:75 challenge dose was subsequently chosen for the main study.

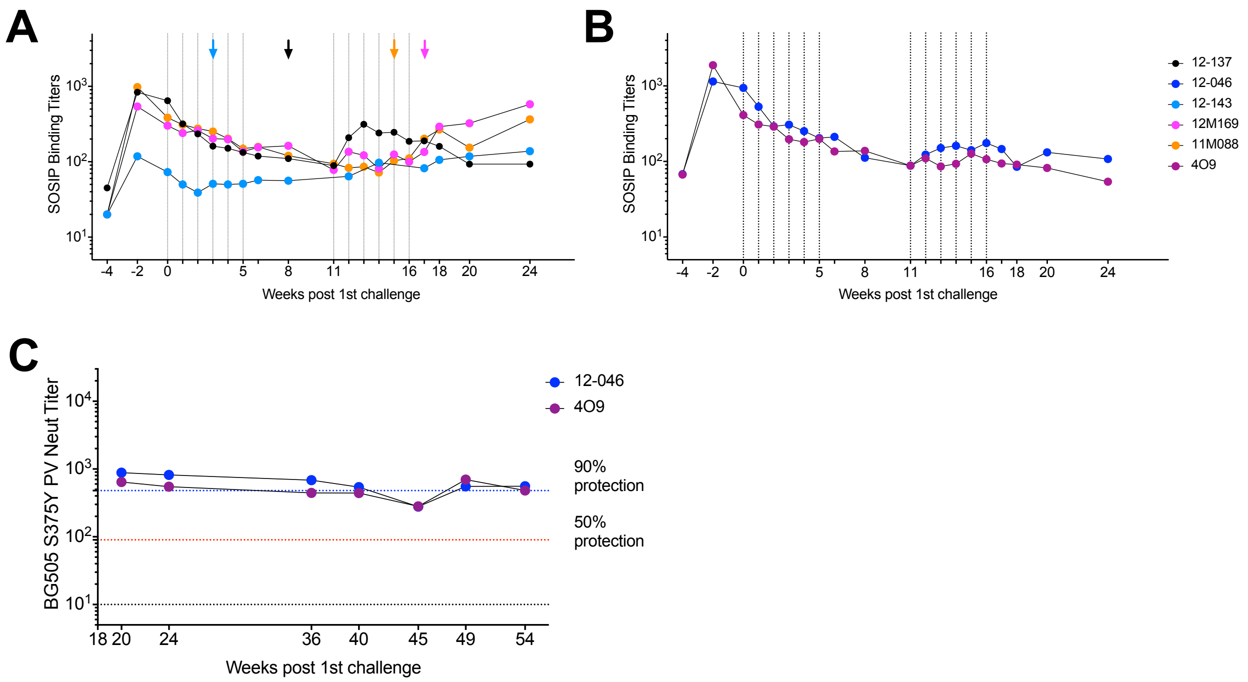

Figure S3, related to Figure 3. NAb titers from protected animals remain stable over one year past immunization (**A-B**) ELISA binding titers in fully protected and infected animals. BG505 SOSIP.664 ELISA EC_{50} binding titers in the high nAb titer group at indicated time points for animals that became infected over the course of 12 challenges (**A**) and fully protected animals (**B**). First detection of plasma viremia is indicated by colored arrows corresponding to the animal IDs shown in the figure legend. (**C**) Longitudinal BG505 S375Y pseudovirus ID_{50} nAb titers in fully protected animals. ID_{50} nAb titers in protected animals remain relatively stable after an initial decline for over 1 year past the final immunization. 90% and 50% protective nAb titer levels as determined in Figure 5C are shown as dotted blue and red lines, respectively.

A

High Titer Group	nAb titer ID50 (ID80) 7d before infection detected	nAb titer ID50 (ID80) at week 20 still uninfected
12-137*	494 (123)	
12-046		958 (239)
12-143	458 (115)	
12M169	141 (35)	
11M088	248 (62)	
4O9		754 (189)
**Geo Mean**	**299 (75)**	**850 (212)**

*14d before infection was detected

Low Titer Group	nAb titer ID50 (ID80) 7d before infection detected	nAb titer ID50 (ID80) at week 20 still uninfected
12-060	110 (27)	
12-153	174 (43)	
12-065	202 (51)	
12-149	57 (< 20)	
12M248	136 (34)	
0Q7	20 (< 20)	
**Geo Mean**	**92 (21)**	

B

High Titer Group	nAb titer ID50 (ID80) 7d before infection detected	nAb titer ID50 (ID80) at week 20 still uninfected
12-137*	26 (< 20)	
12-046		52 (< 20)
12-143	39 (< 20)	
12M169	< 20 (< 20)	
11M088	23 (< 20)	
4O9		40 (< 20)
	**26 (<20)**	**46 (<20)**

*14d before infection was detected

Low Titer Group	nAb titer ID50 (ID80) 7d before infection detected	nAb titer ID50 (ID80) at week 20 still uninfected
12-060	< 20 (< 20)	
12-065	< 20 (< 20)	
12-149	< 20 (< 20)	
12-153	< 20 (< 20)	
12M248	< 20 (< 20)	
0Q7	< 20 (< 20)	
	**< 20** (< 20)	

C

	TZM-bl Neutralization					
	**BG505 Pseudovirus**	**BG505 S375Y Pseudovirus**	**SHIV$_{BG505}$ S375Y Pseudovirus**	**SHIV$_{BG505}$ S375Y IMC (293T-grown)**	**SHIV$_{BG505}$ S375Y rh-CD4 challenge stock**	**ΔSHIV$_{BG505}$ rh-CD4 / 293T**
PGT121	0.01	0.01	0.01	0.01	0.04	3
PGT128	0.004	0.01	0.005	0.01	0.03	3
PGT135	> 5	> 5	> 5	> 5	> 5	
PGT145	0.01	0.003	0.08	0.01	0.06	6
CAP08	0.005	0.003	0.002	0.01	0.005	1
CH03	0.19	0.21	0.34	0.20	1.89	9
VRC01	0.09	0.12	0.19	0.13	0.88	7
b12	> 5	> 5	> 5	> 5	> 5	
PGT151	0.001	0.001	0.001	0.002	0.004	2
4E10	0.99	0.89	0.74	1.54	> 5	<3
10E8	0.07	0.21	0.25	0.14	1.58	12

Figure S4, related to Figure 5. Neutralizing antibody titers 7 days before the detection of viremia. (**A-B**) ID_{50} and ID_{80} neutralization titers of high and low nAb titer animals against BG505 N332 S375Y pseudovirus (**A**), as well as rhCD4+ T-cell-grown SHIV$_{BG505}$ N332 S375Y challenge stock (**B**), 7 days before the detection of viremia in the blood. Titers are averaged from three independent experiments. All assays used TZM-bl target cells. (**C**) Comparison of bnAb IC_{50} neutralization titers (μg/ml) against BG505 N332 pseudovirus, BG505 N332 S375Y pseudovirus, SHIV$_{BG505}$ N332 S375Y pseudovirus and SHIV$_{BG505}$ N332 S375Y infectious molecular clone (IMC) grown in HEK-293T cells (293T-grown), as well as rhCD4+ T-cell-grown (rh-CD4) SHIV$_{BG505}$ N332

S375Y challenge stock. The ratio between the latter two is shown on the right. BG505 N332 S375Y pseudovirus showed similar IC_{50} values as $SHIV_{BG505}$ N332 S375Y IMC grown in HEK-293T cells. Deep sequencing analysis of the challenge stock (see also Figure 4A-B) showed no consensus mutations between the challenge stock and pseudovirus sequence *env* sequences. Conversely, the challenge stock is neutralized to ~100% completion by potent animal sera, indicating that no sizable resistant viral populations exist in the challenge stock. Uncolored ratios between rhCD4+ T-cell and HEK-293T cell-grown $SHIV_{BG505}$ are shown in the last column.

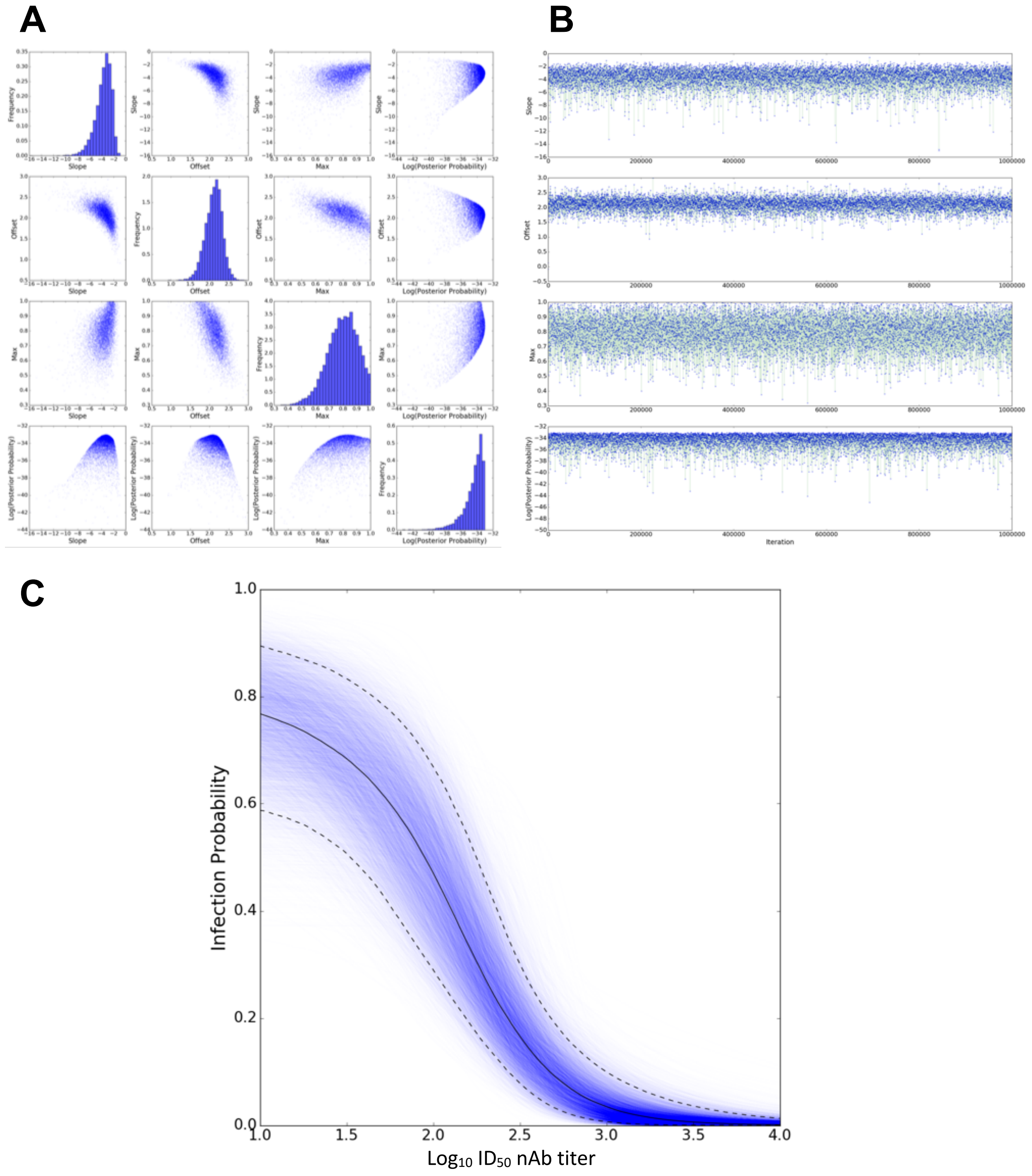

Figure S5, related to Figure 5. Bayesian logistical regression of serum nAb titer and infection probablility. (A) Pairwise scatter plots and histograms of 10,000 samples from the posterior distribution of our modified Bayesian logistic regression model. (**B**) Markov Chain Monte Carlo (MCMC) trace plots for the slope, offset and max parameters of our modified Bayesian logistic regression model, showing rapid mixing and high effective sample sizes (ESSs) above 20,000, indicating strong chain convergence and reliable

posterior inference. (**C**) 10,000 logistic curves were drawn from the posterior distribution of our Bayesian infection probability model (thin blue lines), and 5%, median, and 95% credible intervals computed from these posterior samples. This graph depicts the full extent of the model's uncertainty in the infection probability given the logarithmic ID_{50} BG505 N332 S375Y pseudovirus nAb titer.

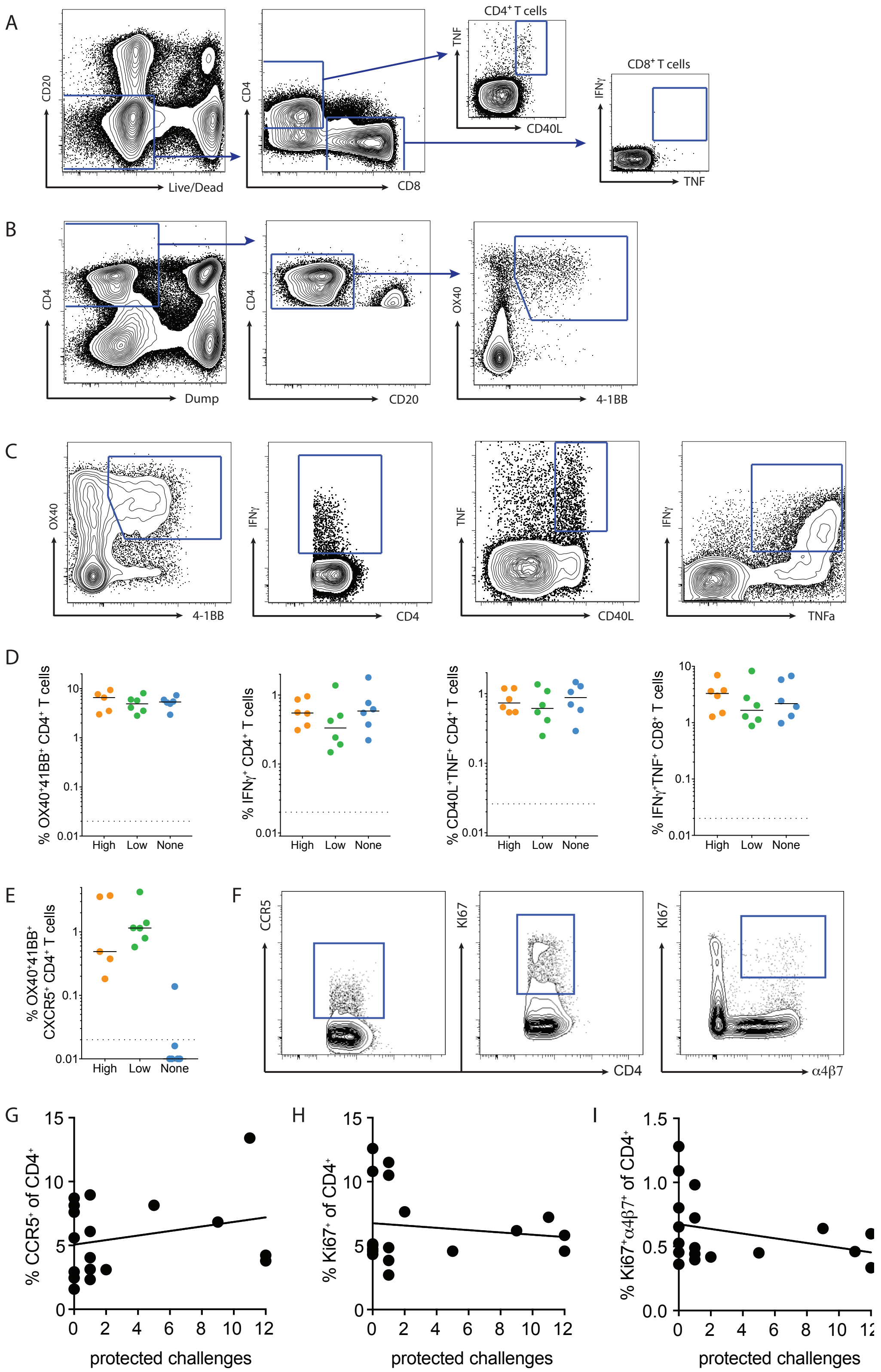

Figure S6, related to Figure 6. Additional analyses of CD4$^+$ and CD8$^+$ T cell activation at week 0 (day of the first challenge). (**A**) Gating strategy for ICS cytokine

expression by $CD4^+$ and $CD8^+$ T cells. (**B**) Gating strategy for detecting Env-specific $CD4^+$ T cells via the OX40/4-1BB AIM assay. Dump includes Live/Dead Viability Dye, CD14, and CD16. (**C**) Representative flow plots of the SEB-stimulated positive control for the $CD4^+$ T cell AIM assay, the $CD4^+$ T cell ICS assays, and the $CD8^+$ T cell ICS assay. (**D**) Quantification of the SEB response for each of the assays in (**C**). Signal from the unstimulated condition was subtracted from the SEB signal for each sample. (**E**) Quantification of the percent of $CXCR5^+$ $CD4^+$ T cells that express OX40 and 4-1BB following antigen stimulation in the $CD4^+$ T cell assay (unstimulated condition subtracted). (**F**) Representative flow plots of CCR5, Ki67, and α4β7 expression on $CD4^+$ T cells from PBMC. (**G-I**) Correlations between the percent of $CD4^+$ T cells that express CCR5 (**G**), Ki67 (**H**), or Ki67 and α4β7 (**I**) with the number of challenges against which the animal was successfully protected.

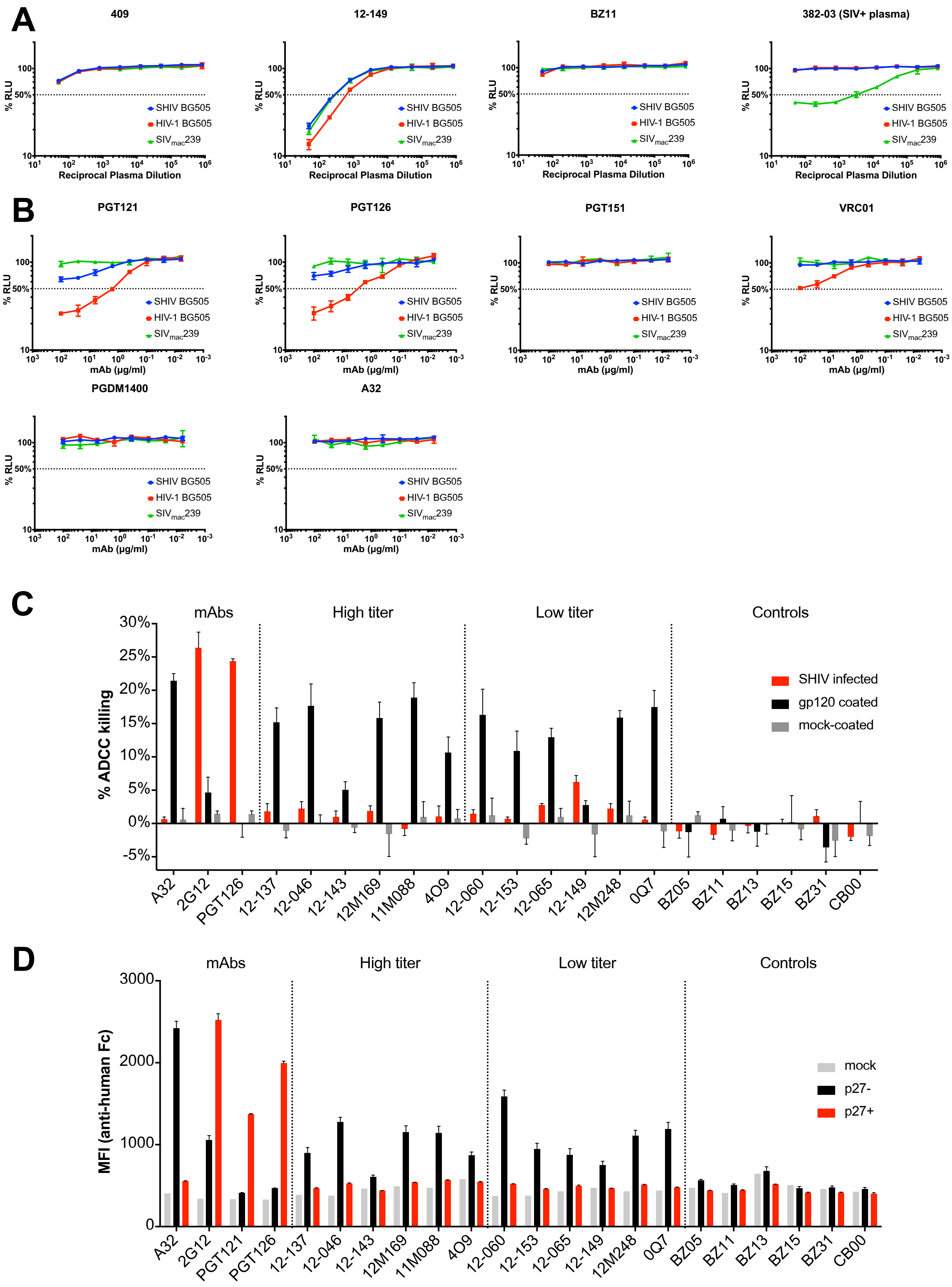

A
409
12-149
BZ11
382-03 (SIV+ plasma)
% RLU
50%
Reciprocal Plasma Dilution
SHIV BG505
HIV-1 BG505
SIVmac239
B
PGT121
PGT126
PGT151
VRC01
PGDM1400
A32
mAb (µg/ml)
C
mAbs
High titer
Low titer
Controls
% ADCC killing
SHIV infected
gp120 coated
mock-coated
A32
2G12
PGT126
12-137
12-046
12-143
12M169
11M088
4O9
12-060
12-153
12-065
12-149
12M248
0Q7
BZ05
BZ11
BZ13
BZ15
BZ31
CB00
D
MFI (anti-human Fc)
mock
p27-
p27+
PGT121

Figure S7, related to Figure 7. Additional analysis of ADCC activity from control mAbs and animal sera at week 0 (day of the first challenge). (**A**) To control for strain specificity of ADCC activity, all immunized animals were tested against $SHIV_{BG505}$ S375Y, HIV-1 BG505 and SIV_{mac}239-infected CEM cells. Shown is the serum ADCC activity from a representative high nAb titer group animal (4O9), the only immunized animal that showed nonspecific ADCC activity (12-149), a representative unimmunized animal (BZ11) as well as serum from a SIV_{mac}239 infected control animal from an unrelated study (382-03). **(B)** As additional positive and negative controls for ADCC activity, a panel of bnAbs and A32, a cluster A specific non-neutralizing antibody, were evaluated as shown. BnAbs showed differing abilities to mediate ADCC against SHIV and SIV-infected cells but showed no activity against SIVmac239-infected cells. The nnAb A32 did not mediate ADCC against HIV or SHIV-infected cells (Bredow et al., 2016; Ding et al., 2016)**,** in line with its inability to bind intact Env trimers. (**C-D**) ADCC measurement and Env-staining of gp120-coated or SHIV infected CEM.NKR cells using mAbs and macaque sera from week 0 (day of the first challenge). (**C**) Comparison of flow cytometry-assessed ADCC killing of CEM.NKR target cells that were either infected with the $SHIV_{BG505}$ S375Y challenge stock (SHIV infected), coated with BG505 gp120 (gp120 coated) or uninfected/uncoated (mock-coated). (**D**) Mean fluorescent intensity (MFI) of $SHIV_{BG505}$ S375Y challenge stock infected or uninfected (mock) CEM.NKR target cells stained with indicated mAbs and sera. $SHIV_{BG505}$ S375Y exposed target cells were gated for productively infected cells (p27+) and uninfected cells (p27-). While p27+ target cells showed little specific staining by animal sera, p27- cells showed various levels of serum staining, indicating gp120 coating of uninfected bystander cells by shed gp120 molecules from productively infected cells. Strong binding by CD4-induced specific mAb A32 to p27-, but not p27+ cells, confirms that uninfected bystander T cells bind shed gp120 via CD4 and thus present gp120 in the CD4-bound state, which is only weakly bound by PGT126. All assays used animal sera diluted 1:250 or mAbs at 5μg/ml. All panels show an average of three independent experiments.

Table S1, related to Figure 1: Genotyping of macaques used in the immunization study. All animals were genotyped for known Mamu (A*01, B*08, B*17) and TRIM-5α alleles (TFP, Q) associated with host restriction of certain SIV strains in nonhuman primates. However, $SHIV_{BG505}$ S375Y uses the SIVmac251-derived SIVmac766 sequence as its genomic backbone to enable replication in macaques (Li et al., 2016). SIVmac251 was shown to be unaffected by otherwise restrictive or partially restrictive TRIM-5α genotypes (TFP/TFP, TFP/Q) *in vivo* (Fenizia et al., 2011). Moreover, SIVmac766 has the identical N-terminal p27 capsid sequence as SIVmac239 (87-APQQGQLREPSGSDIAGTT-SSVDEQIQW-113), which was shown to confer resistance to TRIM-mediated restriction in cell culture experiments (Kirmaier et al., 2010). Consistent with these reports, we observed no delayed onset or reduction of peak viremia in TRIM-5$\alpha^{TFP/TFP}$ homozygous control animals (see for example Figure S2).

Group	Animal ID	Gender	DOB	A*01	B*08	B*17	TRIM	Alleles
High Titer Group	12-137	Female	6/16/12	(-)	(-)	(-)	1-5/6-11	TFP/Q
	12-046	Male	4/1/12	(-)	(-)	(-)	6-11/6-11	Q/Q
	12-143	Female	8/28/12	(-)	(-)	(-)	6-11/6-11	Q/Q
	12M169	Female	5/5/12	(-)	(-)	(-)	1-5/6-11	TFP/Q
	11M088	Female	4/23/11	(-)	(-)	(-)	1-5/6-11	TFP/Q
	4O9	Female	10/1/12	(-)	(-)	(-)	1-5/6-11	TFP/Q
Low Titer Group	12-060	Male	5/26/12	(-)	(-)	(+)	1-5/6-11	TFP/Q
	12-153	Female	10/4/12	(-)	(-)	(-)	1-5/6-11	TFP/Q
	12-065	Female	4/15/12	(-)	(-)	(+)	1-5/6-11	TFP/Q
	12-149	Female	10/1/12	(+)	(-)	(-)	1-5/6-11	TFP/Q
	12M248	Female	6/7/12	(-)	(-)	(-)	1-5/6-11	TFP/Q
	0Q7	Female	9/14/12	(-)	(+)	(-)	6-11/6-11	Q/Q
Unimmunized (Concurrent)	BZ05	Male	6/10/12	(-)	(-)	(-)	1-5/1-5	TFP/TFP
	BZ11	Male	6/11/12	(+)	(-)	(-)	1-5/6-11	TFP/Q
	BZ13	Female	3/21/12	(+)	(-)	(-)	1-5/6-11	TFP/Q
	BZ14	Female	6/4/12	(-)	(-)	(-)	1-5/6-11	TFP/Q
	BZ15	Male	6/18/12	(-)	(-)	(-)	1-5/6-11	TFP/Q
	BZ31	Male	4/28/12	(-)	(-)	(-)	1-5/6-11	TFP/Q
	CB00	Male	4/12/12	(-)	(-)	(-)	6-11/6-11	Q/Q
Unimmunized (1:75 Titration)	BZ01	Female	5/15/12	(+)	(-)	(-)	6-11/6-11	Q/Q
	BZ03	Female	5/19/12	(+)	(-)	(-)	1-5/6-11	TFP/Q
	BZ04	Male	3/26/12	(-)	(+)	(-)	1-5/1-5	TFP/TFP
	A12R056	Male	7/1/12	(-)	(-)	(-)	1-5/1-5	TFP/TFP
	A11L120	Male	6/20/12	(-)	(-)	(-)	1-5/6-11	TFP/Q
	A12L103	Male	6/12/12	(-)	(+)	(-)	1-5/1-5	TFP/TFP
Unimmunized (1:20 Titration)	CB04	Male	7/25/12	(-)	(-)	(-)	1-5/1-5	TFP/TFP
	IQ4	Female	9/9/12	(-)	(-)	(-)	1-5/1-5	TFP/TFP
	A12L105	Female	6/14/12	(+)	(-)	(-)	6-11/6-11	Q/Q
	A12L130	Male	7/21/12	(-)	(-)	(-)	1-5/6-11	TFP/Q
	A12L126	Male	7/11/12	(-)	(-)	(-)	1-5/6-11	TFP/Q
	A12R032	Male	6/3/12	(-)	(-)	(-)	1-5/6-11	TFP/Q

Table S2, related to Figure 2: Estimated frequency of MOI in macaques following SHIV challenge. Estimated frequency (number per 100 animals) of indicated multiplicities of infection (MOIs) as a function of the chosen animal infectious dose (AID) for all challenged animals (upper table) or infected animals only (lower table). MOI frequencies are calculated using the following function: f(MOI) = 100 * dpois(MOI, -ln(1-AID/100)), where dpois is the probability density function for the Poisson distribution, AID is the AID number, e.g. 50 for an AID_{50}, and ln is the natural logarithm function. For an AID_{75} that was used in this study, following a single challenge, 35% of animals are estimated to be productively infected with 1 virion (MOI = 1), 24% with 2 virions (MOI = 2) and 16% with 3 or more virions (MOI > 2). Thus, among infected animals, 35 of 75 animals, or 46%, are expected to be productively infected with 1 virion, 32% with 2 virions, and 22% with 3 or more virions. The observed MOI-distributions for HIV-1 infection in human cohorts do not strictly follow a Poisson distribution of infrequent independent events, due to heterogeneity in MOI-affecting transmission risk-factors (e.g. integrity of genital mucosae) that cannot be controlled for in human cohorts (Abrahams et al., 2009). However, it is reasonable to apply the above method to estimate MOI-distributions in the setting of controlled atraumatic intrarectal challenge of macaques with SHIVs, where observed MOI-distributions do approximate a Poisson distribution (Liu et al., 2010).

Estimated Frequency of Multiplicity of Infection (MOI) - Total Animals

	0	1	2	3	4	5	6	7	8	9	10
AID_{99}	1	5	11	16	19	17	13	9	5	3	1
AID_{90}	10	23	27	20	12	5	2	1	0	0	0
AID_{75}	25	35	24	11	4	1	0	0	0	0	0
AID_{50}	50	35	12	3	0	0	0	0	0	0	0
AID_{25}	75	22	3	0	0	0	0	0	0	0	0
AID_{10}	90	9	0	0	0	0	0	0	0	0	0

Estimated Frequency of Multiplicity of Infection (MOI) - Infected Animals

	1	2	3	4	5	6	7	8	9	10
AID_{99}	5	11	16	19	17	13	9	5	3	1
AID_{90}	26	29	23	13	6	2	1	0	0	0
AID_{75}	46	32	15	5	1	0	0	0	0	0
AID_{50}	69	24	6	1	0	0	0	0	0	0
AID_{25}	86	12	1	0	0	0	0	0	0	0
AID_{10}	95	5	0	0	0	0	0	0	0	0

Table S3, related to Figure 2. Survival models and statistical end point analyses. Statistical analysis of survival model and end point parameters for all animals after 6 consecutive challenges at week 8 (upper table) or following all 12 challenges, including a 5-week resting period between challenge sets, at week 20 (lower table). All indicated tests were calculated in Graphpad PRISM v7.0a.

	**Survival Model (Kaplan-Meier)**			**End point contingency analysis**		
	P-Value vs Controls[1]	P-Value vs Controls[2]	Median Protection	Complete Protection	P-Value vs Control[3]	P-Value vs Control[4]
High Titer (N=6)	0.0002 ***	<0.0001 ****	-	67%	0.0013 **	0.0049 **
Low Titer (N=6)	0.0967	0.0967	2 challenges	0%	-	>0.99
Controls (N=12)	N/A	N/A	1 challange	0%	N/A	N/A
	[1]Gehan-Breslow-Wilcoxon test; [2]Log-rank (Mantel-Cox) test			[3]Chi-square test; [4]Fisher's Exact test		

	**Survival Model (Kaplan-Meier)**			**End point contingency analysis**		
	P-Value vs Controls[1]	P-Value vs Controls[2]	Median Protection	Complete Protection	P-Value vs Control[3]	P-Value vs Control[4]
High Titer (N=6)	0.0002 ***	<0.0001 ****	11 challenges	33%	0.0339 *	0.098
Low Titer (N=6)	0.0967	0.0967	2 challenges	0%	-	>0.99
Controls (N=12)	N/A	N/A	1 challange	0%	N/A	N/A
	[1]Gehan-Breslow-Wilcoxon test; [2]Log-rank (Mantel-Cox) test			[3]Chi-square test; [4]Fisher's Exact test		
