# Supplementary material for: Vaccine-Induced Protection from Homologous Tier 2 SHIV Challenge in Nonhuman Primates Depends on Serum-Neutralizing Antibody Titers
Source: Immunity. 2019 Jan 15;50(1):241–252.e6. doi: 10.1016/j.immuni.2018.11.011 (PMC6335502; doi:10.1016/j.immuni.2018.11.011)
Supplement: Document S2. Article plus Supplemental Information [file mmc2.pdf]

Immunity

Article

Vaccine-Induced Protection from Homologous Tier 2 SHIV Challenge in Nonhuman Primates Depends on Serum-Neutralizing Antibody Titers

Graphical Abstract

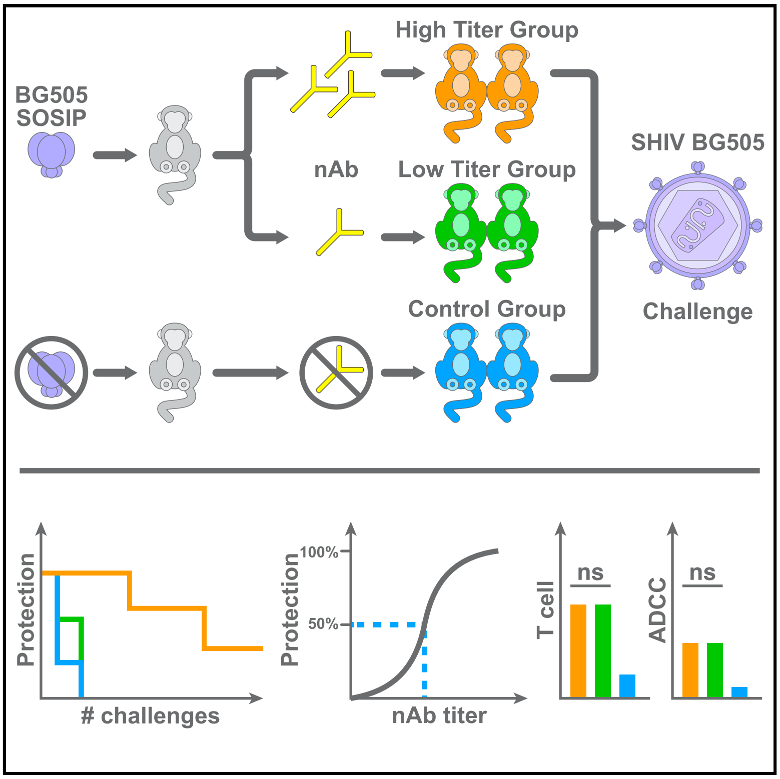

Highlights

- HIV Env trimer immunization protects from autologous tier 2 challenge in macaques
- Neutralizing antibody (nAb) titers correlate with protection and peak viremia
- T cell and ADCC functions do not correlate with protection
- nAb titers of ~1:500 (pseudovirus)/~1:30 (live virus) required for ~90% protection

Authors

Matthias G. Pauthner, Joseph P. Nkolola, Colin Havenar-Daughton, ..., George M. Shaw, Dan H. Barouch, Dennis R. Burton

Correspondence

shane@lji.org (S.C.), shawg@pennmedicine.upenn.edu (G.M.S.), dbarouch@bidmc.harvard.edu (D.H.B.), burton@scripps.edu (D.R.B.)

In Brief

Vaccine-induced immunity to HIV is a major research focus, but relevant correlates of protection remain controversial. Here, Pauthner et al. study protection from hard-to-neutralize $SHIV_{BG505}$ challenge after immunization with native-like BG505 Envelope trimers in nonhuman primates and identify neutralizing antibodies, but not T cell or ADCC activity, as correlates of protection.

Pauthner et al., 2019, Immunity *50*, 241–252
January 15, 2019 © 2018 The Author(s). Published by Elsevier Inc.
https://doi.org/10.1016/j.immuni.2018.11.011

CellPress

CellPress

Vaccine-Induced Protection from Homologous Tier 2 SHIV Challenge in Nonhuman Primates Depends on Serum-Neutralizing Antibody Titers

Matthias G. Pauthner,[1,2,3,20] Joseph P. Nkolola,[4,20] Colin Havenar-Daughton,[2,5,21] Ben Murrell,[6,21] Samantha M. Reiss,[2,5,21] Raiza Bastidas,[1,2,3] Jérémie Prévost,[7,8] Rebecca Nedellec,[1,2,3] Benjamin von Bredow,[9] Peter Abbink,[4] Christopher A. Cottrell,[2,3,10] Daniel W. Kulp,[11] Talar Tokatlian,[2,12] Bartek Nogal,[2,3,10] Matteo Bianchi,[1,2,3] Hui Li,[13] Jeong Hyun Lee,[2,5] Salvatore T. Butera,[1,2] David T. Evans,[9] Lars Hangartner,[1,2,3] Andrés Finzi,[7,8,14] Ian A. Wilson,[2,3,10] Richard T. Wyatt,[1,2,3] Darrell J. Irvine,[2,12,15,16,17] William R. Schief,[1,2,3] Andrew B. Ward,[2,3,10] Rogier W. Sanders,[18,19] Shane Crotty,[2,5,6,22,*] George M. Shaw,[13,22,*] Dan H. Barouch,[4,15,20,22,*] and Dennis R. Burton[1,2,3,15,20,22,23,*]

[1]Department of Immunology and Microbiology, The Scripps Research Institute, La Jolla, CA 92037, USA
[2]Center for HIV/AIDS Vaccine Immunology and Immunogen Discovery (CHAVI-ID), The Scripps Research Institute, La Jolla, CA 92037, USA
[3]IAVI Neutralizing Antibody Center and the Collaboration for AIDS Vaccine Discovery (CAVD), The Scripps Research Institute, La Jolla, CA 92037, USA
[4]Center for Virology and Vaccine Research, Beth Israel Deaconess Medical Center, Harvard Medical School, Boston, MA 02215, USA
[5]Division of Vaccine Discovery, La Jolla Institute for Immunology, La Jolla, CA 92037, USA
[6]Division of Infectious Diseases, Department of Medicine, University of California San Diego, La Jolla, CA 92037, USA
[7]Centre de Recherche du CHUM, Montreal, QC H2X 0A9, Canada
[8]Department of Microbiology, Infectious Diseases and Immunology, Université de Montréal, Montreal, QC H2X 0A9, Canada
[9]Department of Pathology and Laboratory Medicine, University of Wisconsin-Madison, Madison, WI 53705, USA
[10]Department of Integrative Structural and Computational Biology and the Skaggs Institute for Chemical Biology, The Scripps Research Institute, La Jolla, CA 92037, USA
[11]Vaccine and Immunotherapy Center, The Wistar Institute, Philadelphia, PA 19104, USA
[12]Koch Institute for Integrative Cancer Research, MIT, Cambridge, MA 02139, USA
[13]Department of Medicine, University of Pennsylvania, Philadelphia, PA 19104, USA
[14]Department of Microbiology and Immunology, McGill University, Montreal, QC H3A 2B4, Canada
[15]Ragon Institute of Massachusetts General Hospital, Massachusetts Institute of Technology, and Harvard University, Cambridge, MA 02139, USA
[16]Howard Hughes Medical Institute, Chevy Chase, MD 20815, USA
[17]Departments of Biological Engineering and Materials Science & Engineering, MIT, Cambridge, MA 02139, USA
[18]Department of Medical Microbiology, Academic Medical Center, University of Amsterdam, 1105 AZ Amsterdam, the Netherlands
[19]Department of Microbiology and Immunology, Weill Medical College of Cornell University, New York, NY 10065, USA
[20]These authors contributed equally
[21]These authors contributed equally
[22]Senior authors
[23]Lead Contact
*Correspondence: shane@lji.org (S.C.), shawg@pennmedicine.upenn.edu (G.M.S.), dbarouch@bidmc.harvard.edu (D.H.B.), burton@scripps.edu (D.R.B.)
https://doi.org/10.1016/j.immuni.2018.11.011

SUMMARY

Passive administration of HIV neutralizing antibodies (nAbs) can protect macaques from hard-to-neutralize (tier 2) chimeric simian-human immunodeficiency virus (SHIV) challenge. However, conditions for nAb-mediated protection after vaccination have not been established. Here, we selected groups of 6 rhesus macaques with either high or low serum nAb titers from a total of 78 animals immunized with recombinant native-like (SOSIP) Env trimers. Repeat intrarectal challenge with homologous tier 2 $SHIV_{BG505}$ led to rapid infection in unimmunized and low-titer animals. High-titer animals, however, demonstrated protection that was gradually lost as nAb titers waned over time. An autologous serum ID_{50} nAb titer of ~1:500 afforded more than 90% protection from medium-dose SHIV infection. In contrast, antibody-dependent cellular cytotoxicity and T cell activity did not correlate with protection. Therefore, Env protein-based vaccination strategies can protect against hard-to-neutralize SHIV challenge in rhesus macaques by inducing tier 2 nAbs, provided appropriate neutralizing titers can be reached and maintained.

INTRODUCTION

Several vaccine strategies are being pursued to stimulate protective immunity against HIV, including those that combine the elicitation of cellular and humoral responses

This is an open access article under the CC BY license (http://creativecommons.org/licenses/by/4.0/).

(Haynes and Burton, 2017; Stephenson et al., 2016). One of the most intensively studied approaches is focused on inducing neutralizing antibodies (nAbs) to the virus. Early pioneering monkey studies showed that DNA gp120-immunization induces nAb responses that can protect against tier 1 virus challenge (Barnett et al., 2008, 2010; Pal et al., 2006). However, tier 1 viruses like $SHIV_{Ba-L}$ and $SHIV_{SF162-P4}$ are easy to neutralize, typically lead to self-limiting infections, and are not considered representative of circulating viruses in the HIV pandemic. Two recent studies investigated vaccine-induced protection from a mixed tier SIVsmE660 swarm and attribute protection, in part, to nAb and other Ab responses (Keele et al., 2017; Roederer et al., 2014). Currently there is no clear evidence of vaccination-induced nAbs providing protection against viruses possessing hard-to-neutralize clinically relevant tier 2 HIV Env in humans or non human primate (NHP) models.

Enthusiasm for the nAb approach arises from the association of nAbs with protection for other viruses (Tomaras and Plotkin, 2017) and the demonstration that passively administered HIV-neutralizing monoclonal antibodies (mAbs) can afford protection in monkey and mouse models of HIV infection (reviewed in Hessell et al., 2018). As HIV does not infect monkeys, HIV-neutralizing mAbs are assessed by their ability to protect against chimeric simian-human immunodeficiency virus (SHIV) challenge in rhesus macaques (*Macaca mulatta*). However, a major problem in establishing vaccine-induced nAb protection in the SHIV-macaque model has been the notorious difficulty in inducing nAbs by immunization. Indeed, induction of broadly neutralizing antibodies (bnAbs) via immunization has thus far been achieved reproducibly only in cows (Sok et al., 2017). However, we recently showed reliable induction of autologous strain-specific nAbs in macaques against a hard-to-neutralize tier 2 HIV isolate through the use of well-ordered and stabilized HIV envelope glycoprotein (Env) SOSIP trimers as immunogens in optimized approaches (Pauthner et al., 2017), building on previous SOSIP immunization studies in NHPs (Havenar-Daughton et al., 2016; Sanders et al., 2015; Torrents de la Peña et al., 2017). To carry out a protection experiment in macaques then requires construction of a SHIV with the same Env sequence as the immunizing trimer. Fortunately, it has recently become possible to reliably generate infectious SHIVs using *env* sequences from most primary tier 2 HIV strains (Del Prete et al., 2017; Li et al., 2016).

Taking advantage of the advances in both trimer-based immunization strategies and SHIV generation, we immunized macaques with SOSIP trimers of the BG505 *env* sequence (de Taeye et al., 2015; Kulp et al., 2017; Torrents de la Peña et al., 2017), induced BG505-specific tier 2 nAbs, and then challenged animals intrarectally with the neutralization-resistant, pathogenic $SHIV_{BG505}$ (Li et al., 2016). We found that protection was critically dependent on the level of serum nAb titers, but not on other antibody parameters such as V3 binding titers, antibody-dependent cellular cytotoxicity (ADCC), or the induction of T cell activity. We determined an approximate titer threshold for vaccine-induced protection that establishes an experimental benchmark for comparison with nAb-based vaccines to HIV-1.

RESULTS

Immunized Macaques Were Grouped into High and Low nAb-Titer Animals

Our goal was to assess the capability of vaccine-elicited tier 2 nAbs to protect from homologous tier 2 challenge with neutralization-resistant, pathogenic $SHIV_{BG505}$ (Li et al., 2016). We previously developed a protocol for the reliable induction of nAbs and immunized 78 NHPs (Pauthner et al., 2017), inducing varying levels of autologous tier 2 nAb titers after three immunizations with native-like BG505 Env trimers (de Taeye et al., 2015; Kulp et al., 2017; Torrents de la Peña et al., 2017). To design a challenge study powered to detect differences between NHPs with either high or low BG505 nAb titers, we selected six NHPs that were among the top neutralizers and carefully matched them as closely as possible, in terms of gender, age, and weight, with six low nAb titer animals that received similar or identical immunogens (Figure S1). We note that none of the protective or viral breakthrough or antibody kinetic effects described below could be associated with a particular immunogen; as will be seen, observed effects are primarily associated with nAb titer. We further enrolled 12 unimmunized control animals into the study. All animals were genotyped for Mamu and TRIM-5α alleles associated with host restriction in non human primates (Table S1).

High and Low nAb Titer Groups Have Significantly Different Serum nAb Titers after a Final Booster Immunization

To identify a challenge dose that reliably infects unimmunized control animals, we performed a pilot study by intrarectally (IR) inoculating two groups of six macaques at weekly intervals with either 0.5×10^8 or 1.4×10^7 virions of the $SHIV_{BG505}$ S375Y challenge virus grown in rhesus $CD4^+$ T cells (Figure S2). For the main study, we selected a challenge dose of 1.4×10^7 virions (1 mL of 1:75 diluted challenge stock), as it infected at least 4/6 animals after the first challenge and the remaining 2 animals after the second challenge in the pilot study. To maximize nAb titer levels in NHPs prior to challenge, high and low nAb titer animals each received a fourth immunization with the previously used immunogens, adjuvanted in a soluble ISCOMs-class saponin (Figure 1A). All NHPs responded with increased autologous nAb titers 2 weeks post-boost. High and low nAb titer animals continued to show significantly different geometric mean ID_{50} titers of 1:3,790 and 1:103 to BG505 S375Y pseudovirus ($p = 0.002$, Figure 1B), respectively. Neutralization titers to rhesus $CD4^+$ T cell grown $SHIV_{BG505}$ S375Y challenge stock were $\sim$30-fold lower, with significantly different geometric mean titers of 1:102 and < 1:10 when tested on TZM-bl target cells ($p = 0.002$, Figure 1C), respectively.

High nAb Titer Group NHPs Were Robustly Protected from Tier 2 SHIV Infection

Four weeks after the booster immunization, all animals received six weekly IR challenges with $SHIV_{BG505}$. To maximize comparability, viral loads for all animals and time points were simultaneously measured at weeks 6 and 20 (Figures 2A–2C). Five out of six concurrent unimmunized control animals were infected after the first challenge and the remaining animal became viremic

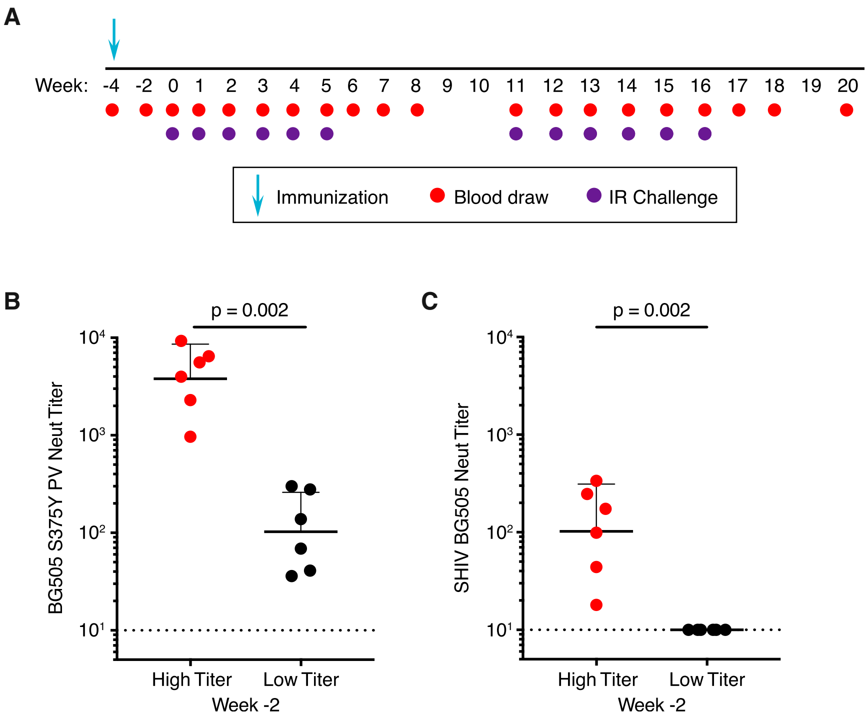

Figure 1. High and Low nAb Titer Group Animals Have Significantly Different Serum nAb Titers after Env Trimer Immunization

(A) Animals, except for the controls, received a booster immunization using the same immunogen that had last been used during the preceding immunization study (Pauthner et al., 2017), typically 100 μg SOSIP trimer adjuvanted in a soluble ISCOMs-class saponin. Intrarectal (IR) challenges with $SHIV_{BG505}$ S375Y commenced 4 weeks thereafter. All groups of animals received six IR challenges starting at week 0. High nAb titer animals that had undetectable serum viral loads at week 6 received a second set of 6 weekly IR challenges starting week 11.

(B and C) Serum neutralizing ID_{50} titers in high and low nAb titer animals at week −2: BG505 S375Y pseudovirus (B) and $rhCD4^+$ T-cell-grown $SHIV_{BG505}$ S375Y challenge stock. (C) Shown are geometric mean titers with geometric standard deviations, significant differences were determined using two-tailed Mann-Whitney U tests.

See also Figure S1.

after the second challenge (Figure 2A). Combined with the unimmunized control NHPs of the dose-matched titration group (Figure S2), at least 9 of 12 unimmunized animals became infected after a single challenge, which approximates to an animal infectious dose of 75% (AID_{75}) (Table S2). Thus, the dose of 1.4×10^7 $SHIV_{BG505}$ virions per IR inoculation employed in this study set a relatively high bar for protection. Unimmunized control animals showed high peak viremia (geometric mean of 5.5×10^6 copies/mL) and consistent set point viral loads in the range of 9.8×10^2 to 4.7×10^4 (geometric mean of 6.2×10^3) at 12 weeks post-infection (Figures 2A, 2E, and S2).

Two out of six low nAb titer animals became infected after the first challenge and the remaining four animals became viremic after the second challenge (Figure 2B), indicating that low nAb titer animals had a possible mild reduction in per-exposure risk compared to unimmunized controls, but the difference was not significant (Figure 2D, Table S3). However, low nAb titer animals had significantly lowered peak viral loads compared to unimmunized controls (1.5×10^5 versus 5.5×10^6 copies/mL) ($p = 0.001$; Figures 2E and 2F).

In contrast, high nAb titer animals showed significant protection from challenge after the first set of challenges at week 8 (Figure 2D, Table S3). Except for macaque 12-143, no animals showed viremia at week 6 and were therefore scheduled to receive a second set of six challenges starting at week 11. The goal of the second challenge set was to assess the duration of protection and to estimate a protective nAb titer threshold as nAb titers declined over time. Over the course of both challenge sets, four initially high nAb titer animals became viremic, after 3, 6, 10, and 12 virus inoculations; however, two animals showed complete sterilizing protection (Figure 2C). In addition, infected high-titer macaques showed significantly lowered peak viremia compared to unimmunized controls (3.2×10^4 versus 5.5×10^6 copies/mL; $p = 0.01$; Figures 2E and 2F), similar to the low nAb titer animals. We theorize that sub-protective levels of serum nAbs at the time of infection, as well as activation of vaccine-induced memory B cells leading to the rapid production of Abs, likely curtail emerging primary viremia, thus reducing peak viral loads.

The protection from infection for high nAb titer animals compared to unimmunized controls after both 6 and 12 challenges was significant ($p < 0.0001$; Figure 2D, Table S3) and animals in this group remained uninfected for a median of 11 challenges (Table S3). It should be emphasized that, for all vaccinated animals, nAb titers declined throughout the challenge schedule, unless animals became infected as detailed below. In this respect, our study distinguishes itself from those in which antibody titers leveled off prior to challenge, as a result of the short 4-week interval here between final immunization and first challenge. However, we deliberately took advantage of declining nAb titers to determine a nAb-mediated threshold of protection. In summary, high nAb titer animals showed protection over the course of multiple challenges, while low nAb titer and unimmunized animals became rapidly infected.

Tier 2 nAb Titers Correlate with Protection

Unimmunized control animals developed BG505 S375Y pseudovirus ID_{50} nAb titers 8–12 weeks after infection in response to $SHIV_{BG505}$ S375Y infection (Figure 3A). By comparison, vaccine-induced nAb titers in low titer animals initially declined, but then began to rise only 1–2 weeks after infection, i.e., much more rapidly than in unimmunized animals (Figure 3B). The early rise of nAb titers after infection of low nAb titer animals is thus likely due to recall responses of BG505 Env immunogen-induced memory B cells. Interestingly, BG505 nAb titers rose to substantially higher ID_{50} titers (3/6 animals > 1:750) than

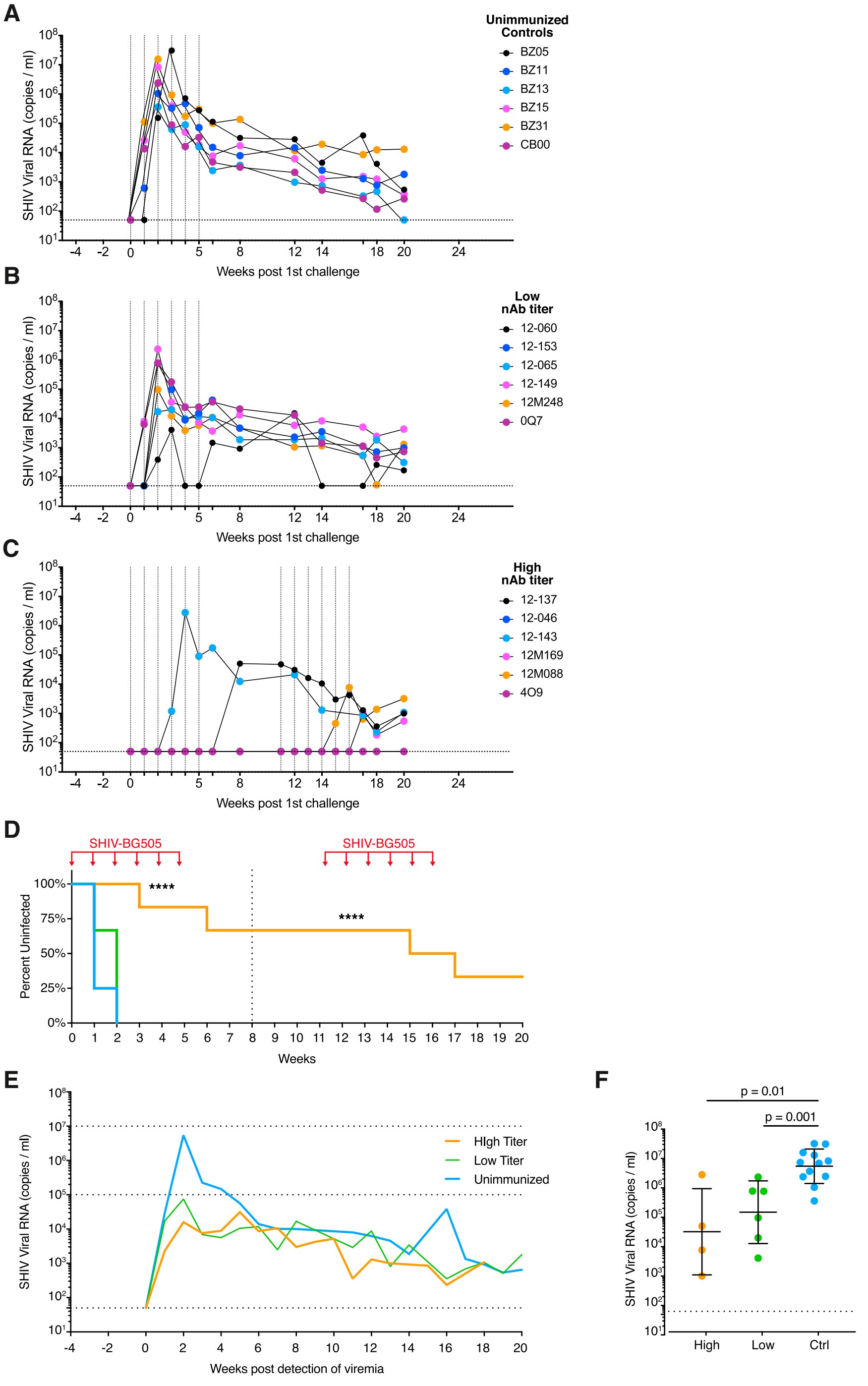

Figure 2. High nAb Titer Animals Show Robust Protection from $SHIV_{BG505}$ Challenge

(A–C) Viral loads of animals throughout the challenge schedule: unimmunized concurrent controls (A), low nAb titer (B), and high nAb titer (C) macaques. IR challenges are indicated with vertical dotted lines. Horizontal dotted lines denote the limit of detection.

(D) Kaplan-Meier curves indicating percent uninfected animals over the duration of the study. Challenge time points are indicated with red arrows. Significance levels are indicated with stars; ****$p < 0.0001$. Statistics were calculated for both the first (dotted line at week 8) and second challenge sets (see Table S3).

(E) Geometric mean viral loads of indicated groups, normalized to the detection of viremia in the blood. Horizontal lines at 10^5 and 10^7 viral RNA copies/mL serve as visual aids.

(F) Comparison of peak viral loads between high nAb titer (High), low nAb titer (Low), and unimmunized (Ctrl) animals. Geometric mean viral loads are shown with geometric standard deviations. Significant differences were determined using two-tailed Mann-Whitney U tests.

See also Figure S2.

previously achieved by four immunizations of these six animals with ISCOMs-adjuvanted BG505 native-like Env trimers (Figures 1B, 3B, and S1B). The marked increases in BG505 nAb titers after infection suggest that outbred macaques that did not respond well to vaccination were not inherently incapable, by genetic or other means, of developing high nAb titer responses, although this conclusion should be caveated by the observation that antigen dose and delivery vary greatly between vaccination and natural infection. Better immunogen presentation and more targeted adjuvants are likely needed to increase the reliability of high nAb titer development and to address current shortcomings in the durability of nAb responses induced by protein-only immunizations (Havenar-Daughton et al., 2017).

High nAb titer animals that became infected showed a comparable increase in BG505 S375Y nAb titers ~1–4 weeks after infection. The only exception was animal 12-137, who suppressed viremia for 3 weeks after challenge at week 5 and thus delayed a surge in nAb titers until week 11 (Figure 3C). Animal 12-143, which became viremic at week 3, showed only a small rise in nAb titers at week 6, suggesting possible rapid viral escape. PacBio sequencing of viral species in 12-143 plasma at week 8 in fact revealed that >95% of sequenced *env* genomes contained putative escape mutations at residues 168 and 192 (Figure 4A). Similarly, *env* genomes in 12-137 plasma at weeks 12 and 16 showed putative escape mutations at residues 354 and 356, flanking the N355 glycan, which coincided with onset of nAb titer decay at week 12 (Figures 4B and 3C). nAb specificities to the N355 region were observed in BG505 SOSIP immunized rabbits (Klasse et al., 2018) and were detected in week 0 plasma of animal 12-137 using electron microscopy-based serum mapping (Figure 4C; Bianchi et al., 2018). Serum neutralization assays revealed that the observed viral point mutations in fact confer neutralization resistance to sera from the respective animals (Figures 4D and 4E). Animals 12M169 and

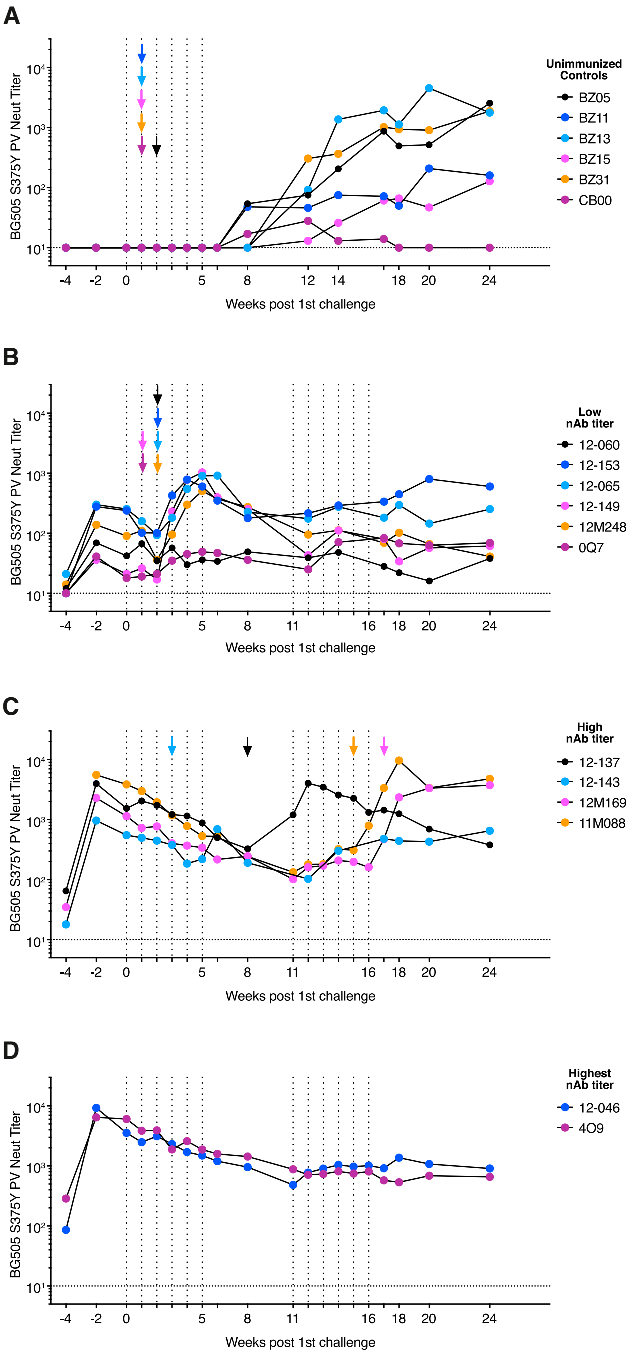

Figure 3. Autologous Tier 2 nAb Titers Surge in Vaccinated Animals after Infection

Serum neutralizing antibody titers throughout the challenge schedule: BG505 S375Y pseudovirus ID_{50} nAb titers rise 8–12 weeks after infection in unimmunized animals (A) or 1–2 weeks after detection of viremia in low nAb titer animals (B). BG505 S375Y pseudovirus ID_{50} nAb titers in macaques that became infected over time (C) or showed sterilizing protection (D). First detection of plasma viremia is indicated by colored arrows corresponding to the animal IDs shown in the respective figure legends. See also Figure S3.

12M088, which become infected at weeks 16 and 14, respectively, exhibited slow declines in vaccine-induced nAb titers which then rose after infection (Figure 3C). The nAb titers of fully protected animals 12-046 and 4O9 (Figure 3D) initially declined and then plateaued at ~1:800 around week 10 and remained stable for the remainder of the study. This trend was mirrored in longitudinal ELISA EC_{50} binding titers (Figures S3A and S3B). Uninfected animals retained robust nAb titer levels more than 1 year after the final immunization (Figure S3C).

The differences in both BG505 S375Y pseudovirus as well as $SHIV_{BG505}$ challenge stock neutralization ID_{50} titers between high and low nAb titer animals at week −2 were, as anticipated, highly significant (Figures 1B and 1C). Peak nAb titers at week −2 accurately predicted the duration of protection, identifying nAb titers as the primary correlate of protection ($p < 0.0001$; Figure 5A). Using the BG505 S375Y pseudovirus assay, a statistically significant difference was found between nAb titers in immunized animals 7 days prior to onset of viremia and animals that remained uninfected until week 20 ($p = 0.03$; Figures 5B and S4A).

To numerically quantify the relationship between BG505 S375Y pseudovirus nAb titers and likelihood of infection, we developed a modified Bayesian logistic regression model using the neutralization and viral load data from all three animal groups (Figures 5C and S5). The posterior median infection probability at the limit of nAb titer detection was 77%, agreeing closely with an estimated animal infectious dose of 75% in unimmunized controls. A median per-challenge infection probability of 50% was attained with ID_{50} titers of 1:90, which agrees well with the often-quoted 50% protective ID_{50} titer of ~1:100, derived from bnAb passive transfer studies (Hessell et al., 2018; Moldt et al., 2012; Parren et al., 2001; Pegu et al., 2014; Shingai et al., 2014), although we note that various different neutralization assays with differing sensitivities were employed in these studies. To achieve an infection probability of 10% (or 90% protection), an ID_{50} titer of 1:476 (CI: 272–991) was required. In agreement with our model, animals with nAb titers above ~1:500 remained protected over all 12 challenges, while animals with nAb titers below 1:200 generally became infected with only 1–2 challenges. For the rhesus $CD4^+$ T cell grown $SHIV_{BG505}$ S375Y virus stock, an ID_{50} titer of ~1:30 (Figure S4B) was the protection threshold. The observed disparity between pseudovirus and PBMC-grown virus assays was relatively large compared with that reported for many mAbs but still within previously observed ranges (Figure S4C; Cohen et al., 2018; Provine et al., 2012). Thus, tier 2 nAb titers both predicted and correlated with protection from infection.

T Cell Activity and Serum Antibody-Dependent Cell-Mediated Cytotoxicity (ADCC) Do Not Correlate with Protection

We further investigated other parameters that may have contributed to protection. Robust Env-specific $CD4^+$ T cell responses were elicited by BG505 Env trimer immunization and were equivalent in magnitude between the high and low nAb titer groups of immunized animals before challenge (Figures 6A, 6E, and S6A–S6C). Cytokine-producing Env-specific $CD4^+$ T cells were also comparable between the two groups of

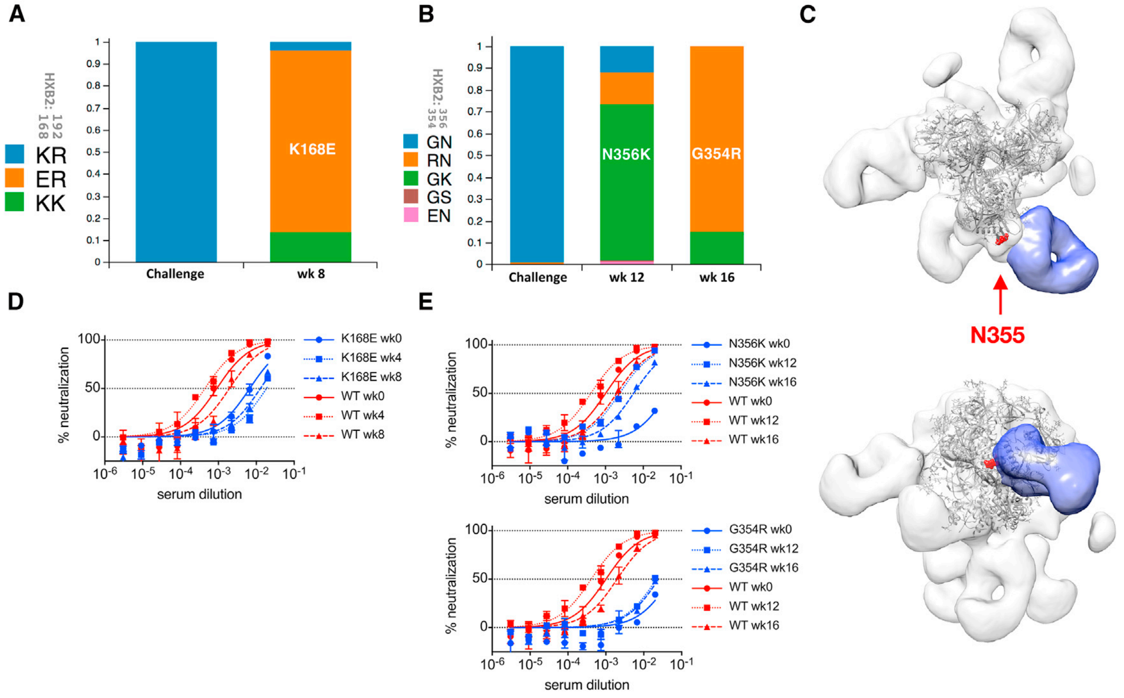

Figure 4. Viral Escape Follows Resurgence of nAb Titers after Infection

(A and B) Viral sequencing after SHIV challenge and infection. PacBio viral sequencing data of two high nAb titer animals, 12-143 (A) and 12-137 (B), indicates putative escape mutations after infection, at the time points indicated. Challenge denotes challenge stock.

(C) Negative stain electron micrograph of a C3-V5 directed serum specificity (purple) observed among cleaved serum Fab fragments of animal 12-137 at week 0 bound to BG505 SOSIP.664. Shown are a top view (upper panel) and a 90° rotated front view (lower panel). N355 is highlighted in red.

(D and E) Serum neutralization data of animals 12-143 (D) and 12-137 (E) at indicated time points against either BG505 S375Y pseudovirus (WT) or indicated mutants thereof. Error bars indicate technical replicates.

immunized animals before challenge (IFN-γ^+, Figures 6B and 6F; TNF$^+$CD40L$^+$, Figures 6C, 6G, and S6D) and protein vaccine-elicited Env-specific CD8$^+$ T cells were undetectable, as expected (Figures 6D and 6H). Thus, Env-specific CD4$^+$ T cells or CD8$^+$ T cells were not a correlate of protection.

Concerns have been raised about vaccine-elicited CD4$^+$ T cell responses enhancing susceptibility to infection by HIV (Fauci et al., 2014; Hu et al., 2014) or SIV (Fouts et al., 2015; Staprans et al., 2004) by providing more targets for infection at the mucosal site of transmission (Bukh et al., 2014; Carnathan et al., 2015; Martins and Watkins, 2018; Qureshi et al., 2012), most likely due to the presence of activated Th1 cells in the mucosa, which was correlated with CCR5, $\alpha4\beta7$, or proliferation in different studies. Minimal Th1 cells were detected in the BG505 Env trimer immunized animals (IFN-γ^+CD4$^+$ T cells; Figures 6B and 6F). CCR5$^+$, Ki67$^+$, or Ki67$^+$/$\alpha4\beta7^+$ CD4$^+$ T cells in peripheral blood prior to challenge were not correlated with susceptibility to infection or protection (Figures S6E–S6H). Thus, we observed robust protection of high nAb titer animals against a mucosal SHIV challenge despite substantial levels of Env-specific vaccine-induced CD4$^+$ T cells in peripheral blood at 4 weeks after the final immunization. The difference in our study may be due to a lack of Th1 cells or mucosal homing CD4$^+$ T cells in response to the protein vaccine, compared to previously used viral vectors (Bukh et al., 2014; Carnathan et al., 2015; Fauci et al., 2014; Hu et al., 2014; Staprans et al., 2004). Alternatively, nAb-mediated protection against HIV/SIV may more readily overcome possible adverse consequences of increased numbers of activated CD4$^+$ T cell targets than the non-neutralizing Abs (nnAbs) raised in the earlier studies.

To investigate possible contributions of ADCC of both nAbs and nnAbs, we tested animal sera in two infection-based assays: SHIV$_{BG505}$-infected CEM.NKR luciferase reporter cells (Figure 7A; Alpert et al., 2012) and flow cytometric analysis of ADCC in p27$^+$ SHIV$_{BG505}$-infected CEM.NKR target cells (Figure 7B; Veillette et al., 2014). Using either assay, we failed to detect meaningful ADCC activity at week 0 with the exception of a single animal, 12-149, which was a low titer animal whose ADCC activity was non-specific and included activity against control SIV$_{mac}$239 (Figure S7A). The absence of observed ADCC activity can be partially explained by the tier 2 character of BG505 Env. In native Env trimer-based ADCC assays, nnAb and tier 1 nAbs fail to mediate ADCC activity against hard-to-neutralize tier 2 HIV isolates, as previously reported (von Bredow et al., 2016; Ding et al., 2015). In addition, ADCC activation in infection-based assays varies strongly depending on the targeted epitope, which is likely related to the Ab binding stoichiometry to the epitope and the ability to cross-link sparse trimers on the virion surface (Figures S7B–S7D; von Bredow et al., 2016; Ding et al., 2015).

Unlike infection-based assays, ADCC killing measured on CEM.NKR target cells coated with BG505 gp120 was robust

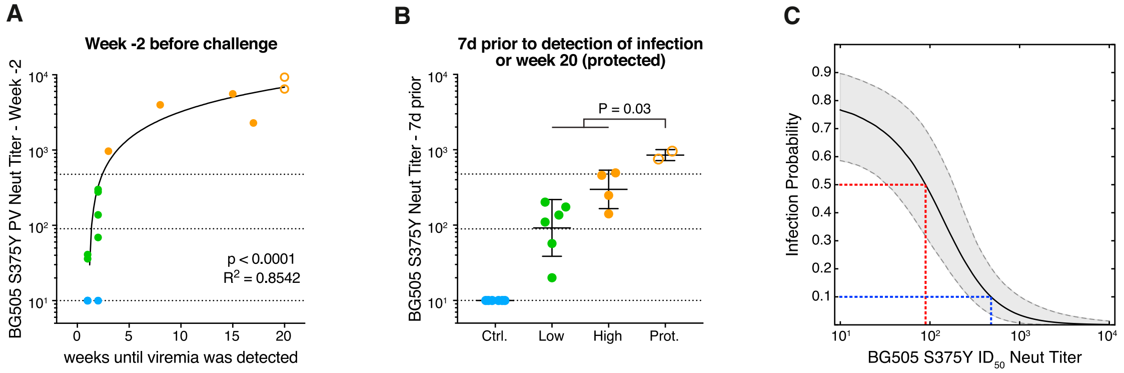

Figure 5. Protection Is Associated with Serum nAb Titers Greater than ~1:500

(A) Correlation of BG505 S375Y pseudovirus ID_{50} nAb titers at week −2 with the number of weeks until viremia was detected.

(B) BG505 S375Y pseudovirus ID_{50} nAb titers of control (Ctrl.), low nAb titer (Low), and high nAb titer (High) animals 7 days before detection of viral load in the blood and at week 20 for protected (Prot.) animals that showed sterilizing protection throughout the study. All nAb titers were measured in TZM-bl assays. Shown are geometric mean titers with geometric standard deviations. Correlations were calculated using Spearman correlation tests, comparisons between groups were calculated using Mann-Whitney U tests. Horizontal lines indicate 50% and 90% protective nAb titers as defined in (C).

(C) The 5%, median, and 95% credible intervals (CI) are shown for the probability of infection in relation to serum BG505 S375Y pseudovirus nAb titer, inferred using a modified Bayesian logistic regression model (see Figure S5). The posterior median infection probability at the limit of nAb titer detection was 77%, corresponding to an AID_{77}. A median infection probability of 50% is attained with an ID_{50} titer of 1:90 (red line, CI: 34-178), and an infection probability of 10% with an ID_{50} titer of 1:476 (blue line, CI: 272-991).

See also Figures S4 and S5.

but did not distinguish between high and low nAb titer animals and, therefore, was not associated with protection (Figure 7C). ADCC killing of gp120-coated cells did correlate with BG505 gp120 binding, indicating that gp120-binding antibodies are sufficient to induce ADCC in this assay (Figure 7D) but cannot mediate ADCC to native membrane-bound Env on infected cells. Thus, ADCC unlikely contributes to protection. We also observed considerable staining of $p27^{-}$ uninfected bystander T cells by both mAbs and animal sera, which appears to result from antibody binding to shed gp120 from infected cells that is captured on CD4 of uninfected cells (Figure S7D; Richard et al., 2018). Overall, these results suggest caution in the use of ADCC assays that are either based on recombinant gp120 or gp140 binding, rather than native Env on virus-infected cells, or cannot distinguish productively infected from uninfected bystander cells (Ackerman et al., 2016; Ferrari et al., 2011; Huang et al., 2016; Johansson et al., 2011; Kristensen et al., 2018). We note that the results pertain to ADCC; there remains the possibility that other Fc-mediated effector functions might contribute to protection.

Lastly, we determined BG505 SOSIP.664 (Figure 7E), V3-peptide (Figure 7F), and BG505 gp120 binding titers (Figure 7G) for all groups at week 0 since V3-targeting antibodies (Balasubramanian et al., 2018), and binding antibodies in general have been associated with anti-viral activities (Excler et al., 2014). No significant differences between high and low neutralizer animals were detected. In summary, neither T cell activity nor ADCC and gp120 antibody binding titers correlated with protection.

DISCUSSION

Vaccine protection against HIV in humans and against SIV and SHIV in macaques has been associated with non-neutralizing antibodies (Barouch et al., 2015; Haynes et al., 2012). Here, we demonstrated that vaccine-induced tier 2 nAbs, but not other antibody parameters such as V3 binding titers, antibody-dependent cellular cytotoxicity (ADCC), or induction of T cell activity, were a correlate of protection from homologous $SHIV_{BG505}$ infection in macaques. We employed a challenge dose of virus corresponding to an AID_{75}, which set a relatively high bar for protection, given that most animals (~53%) in the control arm were estimated to have been productively infected by two or more viruses (Table S2). Similar rates of multivariant virus transmission have been reported in men who have sex with men and injection drug users who acquire HIV-1 infection (38% and 60% with a MOI of 2 or higher, respectively), while heterosexual cohorts show lower multivariant transmission frequencies (~19%). Thus, our model mimics the conditions of productive transmission events, underlining the physiological relevance of the challenge dose that we used (Bar et al., 2010; Li et al., 2010).

We showed, in the model system described, that animals remain protected from SHIV infection in a nAb titer-dependent manner, which suggests a strong relationship between circulating nAb titers in the blood and protection from mucosal challenge with difficult-to-neutralize, tier 2 $SHIV_{BG505}$. At the same time, our data suggest that vaccine protection can occur in the absence of ADCC. We showed that unprotected animals have relatively high levels of ADCC when measured in a widely used ADCC assay that uses target cells coated with monomeric gp120, but not with $SHIV_{BG505}$-infected target cells. We further provided evidence that adjuvanted protein immunization with HIV Env can induce nAb titers that are durable and protective over longer periods of time, if high initial nAb titers after immunization can be reached. This has been a major concern in the HIV vaccine field (Sundling et al., 2013), but also for other protein-based vaccines, such as recombinant

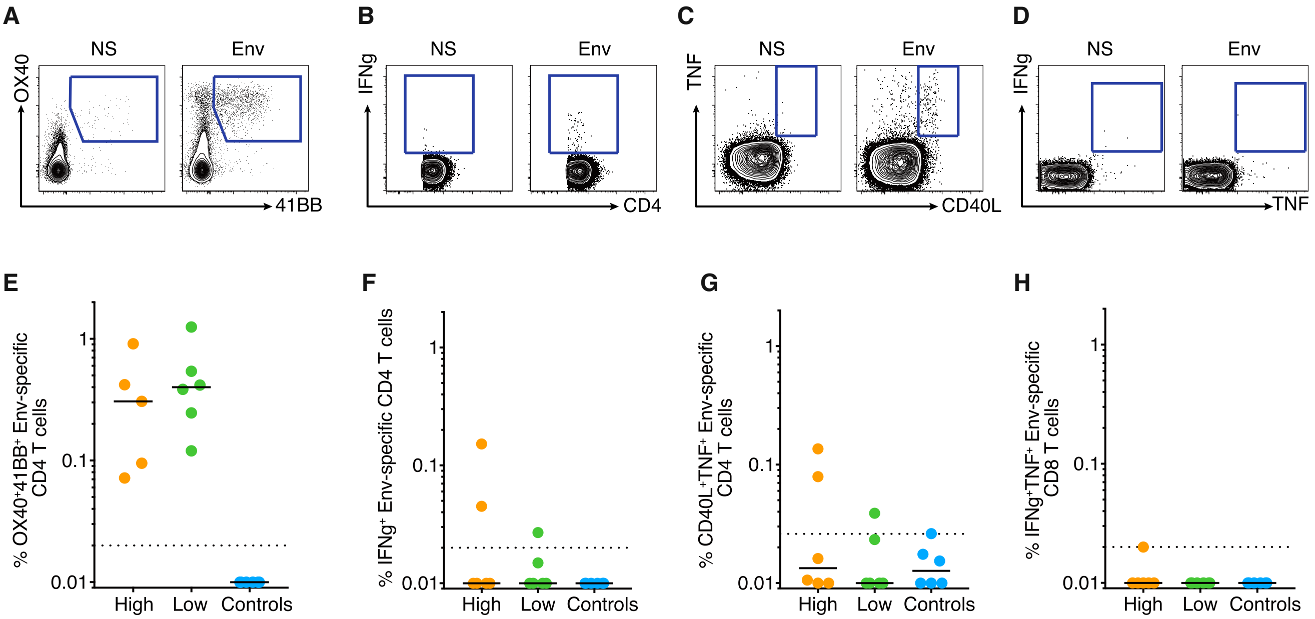

Figure 6. HIV Env-Specific CD4+ T Cells and Env-Specific CD8+ T Cells at Week 0 Are Not Associated with the Observed Protection from Infection

(A–C) Representative flow plots of Env-specific CD4+ T cells from week 0 PBMCs: using an OX40/4-1BB AIM assay (Reiss et al., 2017) (A), intracellular staining (ICS) assay for IFN-γ (B), and ICS assay for TNF/CD40L (C) when not stimulated (NS) versus stimulated with antigen (Env).

(D) Representative flow plot of IFN-γ and TNF expression in CD8+ T cells by ICS when not stimulated (NS) versus stimulated with antigen (Env).

(E–G) Quantification of the percent of CD4+ T cells that are Env-specific based on: OX40/4-1BB (E), IFN-γ (F), or CD40L/TNF (G) expression.

(H) Quantification of the percent of CD8+ T cells that are Env-specific based on IFN-γ and TNF expression. Signal from the unstimulated condition was subtracted from the antigen-specific signal for each sample. Each dot represents an individual animal. Shown are high and low nAb titer, as well as control group animals. See also Figure S6.

influenza vaccines (Krammer and Palese, 2015). Importantly, we identified that a serum ID_{50} nAb titer of ~1:500 against the homologous BG505 S375Y pseudovirus at the time of challenge can confer reliable protection of >90%, meaning that 9 of 10 challenges with a physiologically relevant AID_{75} dose would not result in infection. Finally, protection was observed for polyclonal neutralizing Ab responses that, as above and earlier (Pauthner et al., 2017), target multiple specificities on Env and not simply the previously described glycan hole on BG505 Env (McCoy et al., 2016).

In conclusion, we provide evidence that protein immunization with native-like Env trimers can induce potent and protective nAb titers in the SHIV/macaque model. Thus, nAb-mediated protection from tier 2 virus challenge is not limited to bnAbs, which are generally focused to a single site of vulnerability and have a defined effector-function profile, but can also be accomplished by polyclonal autologous nAb responses of sufficient magnitude and specificity. The latter, by comparison, comprise a broad range of neutralizing and non-neutralizing antibody lineages to various, often overlapping epitopes that interact both synergistically and competitively (Klasse et al., 2018; Pauthner et al., 2017; Sanders et al., 2015; Torrents de la Peña et al., 2018). The protective nAb titer threshold against the homologous challenge virus that we determined is in rough accord with passive antibody transfer studies and provides a benchmark for comparison with upcoming antibody protection studies against HIV in humans (http://ampstudy.org).

STAR★METHODS

Detailed methods are provided in the online version of this paper and include the following:

- KEY RESOURCES TABLE
- CONTACT FOR REAGENT AND RESOURCE SHARING
- EXPERIMENTAL MODEL AND SUBJECT DETAILS
- METHOD DETAILS
  - Rhesus monkey immunizations and challenge
  - Viral Load Assay
  - Serum neutralization assays
  - Serum binding ELISAs
  - ADCC assays
  - T cell analysis
  - Full length *env* viral sequencing
  - Complex preparation for negative-stain EM
  - Negative-stain EM
- QUANTIFICATION AND STATISTICAL ANALYSIS

SUPPLEMENTAL INFORMATION

Supplemental Information includes seven figures and three tables and can be found online with the article at https://doi.org/10.1016/j.immuni.2018.11.011.

ACKNOWLEDGMENTS

We would like to thank Laura Pruyn and Joan Allmaras for excellent administrative support. The funding for this study was provided by NIAID

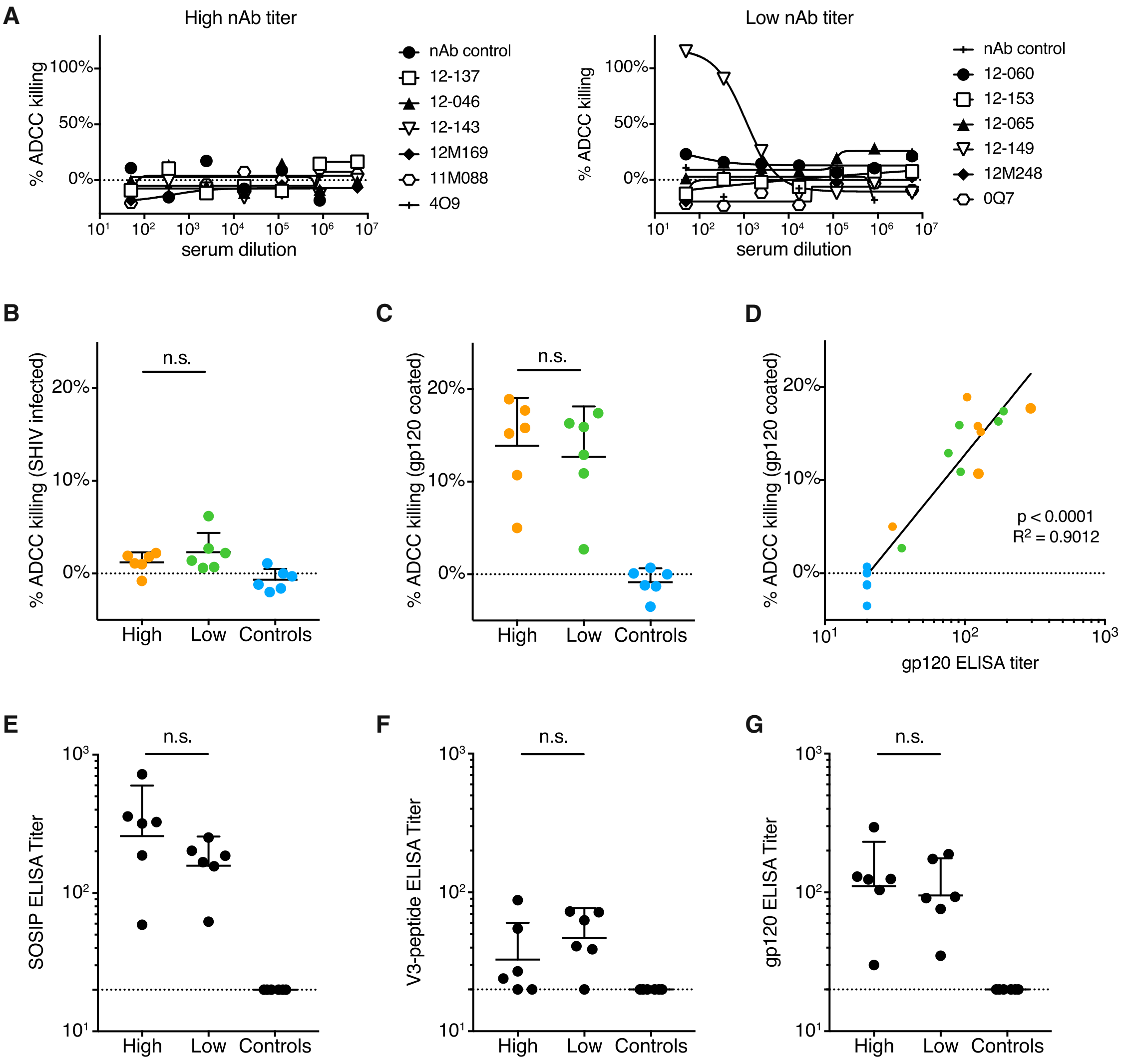

Figure 7. ADCC Activity at Week 0 Measured in SHIV Infection as well as gp120-Based Assays Is Not Associated with the Observed Protection from Infection

(A–D) ADCC activity from sera of high and low nAb titer as well as control animals at week 0. ADCC activity in titrated sera was measured using $SHIV_{BG505}$ challenge stock infected CEM.NKR luciferase-reporter target cells and CD16 transfected KHYG-1 effector cells (A) or in 1:250 diluted sera by flow cytometric analysis of ADCC activity in either $p27^{+}$ $SHIV_{BG505}$-infected CEM.NKR cells (B) or BG505 g120-coated CEM.NKR cells (C), using PBMCs as effector cells. Shown are means with standard deviations. ADCC activity in BG505 gp120-coated CEM.NKR cells correlated with BG505 gp120 binding titers (D).

(E–G) ELISA EC_{50} binding titers at week 0 to: BG505 SOSIP.664 (E), BG505 V3-peptide (F), or BG505 gp120 (G). Sera from high and low nAb titer animals, as well as unimmunized control animals were tested for ELISA binding titers at week 0. Shown are geometric mean titers with geometric standard deviations. Correlations were calculated using Spearman correlation tests, comparisons between groups were calculated using two-tailed Mann-Whitney U tests.

See also Figure S7.

UM1AI100663 (Center for HIV/AIDS Vaccine Immunology and Immunogen Discovery). The Bill & Melinda Gates Foundation (OPP1084519) and the International AIDS Vaccine Initiative helped support the design of some immunogens used in this study. This work was further supported by the National Institutes of Health grants AI121135 (D.T.E.), AI136621 (A.B.W.), AI124377, AI126603, AI128751 (D.H.B.), and OD011106 (Wisconsin National Primate Research Center) and grant OPP1145046 from the Bill & Melinda Gates Foundation (G.M.S.). A.F.'s work was supported by CIHR foundation grant #352417. A.F. is the recipient of a Canada Research Chair on Retroviral Entry RCHS0235. J.P. is the recipient of a CIHR Fellowship Award. B.M. was supported by grants R00AI120851 and UM1AI068618 from the National Institute of Allergy and Infectious Diseases. PacBio SMRT sequencing was performed with the support of the Translational Virology Core at the UC San Diego Center for AIDS Research (P30 AI036214) and the IGM Genomics Center, University of California, San Diego, La Jolla, California. C.A.C. is supported by a NIH F31 Ruth L. Kirschstein Predoctoral Award AI131873 and by the Achievement Rewards for College Scientists Foundation.

AUTHOR CONTRIBUTIONS

The TSRI CHAVI-ID immunogen working group consisting of S.C., W.R.S., A.B.W., I.A.W., R.T.W., and D.R.B. (with the assistance of M.G.P., S.T.B., G.M.S., and D.H.B.) designed the challenge study and laid out the experimental strategy. J.P.N. and D.H.B. oversaw all rhesus macaque immunizations and challenges, including sample acquisition, processing, storage, and distribution. P.A. and D.H.B. performed and oversaw viral load assays. H.L. and

G.M.S. designed and produced the SHIVBG505 challenge stock. C.A.C., D.W.K., and T.T. with oversight from D.J.I., A.B.W., W.R.S., and R.W.S. designed and produced the boosting immunogens for the study. M.G.P., R.B., J.H.L., and D.R.B. designed HIV pseudovirus mutants and performed and oversaw neutralization experiments as well as ELISA binding experiments. C.H.-D., S.M.R., and S.C. performed and oversaw flow cytometric analysis of T cell activation. J.P., R.N., and B.v.B. with oversight from D.T.E., L.H., and A.F. performed ADCC assays. B.N. and M.B. with oversight from L.H. and A.B.W. performed serum negative-stain EM analysis. B.M. and M.G.P. performed statistical analysis of data sets. M.G.P., J.P.N., C.H.-D., B.M., S.M.R., J.P., R.N., B.v.B., T.T., S.T.B., D.T.E., L.H., A.F., I.A.W., R.T.W., D.J.I., W.R.S., A.B.W., R.W.S., S.C., G.M.S., D.H.B., and D.R.B. analyzed data sets and contributed edits to the manuscript. M.G.P., S.C., and D.R.B. wrote the manuscript.

DECLARATION OF INTERESTS

The authors declare no competing interests

Received: August 27, 2018
Revised: September 21, 2018
Accepted: November 5, 2018
Published: December 11, 2018

SUPPORTING CITATIONS

The following references appear in the Supplemental Information: Abrahams et al. (2009); Fenizia et al. (2011); Kirmaier et al. (2010); Liu et al. (2010).

REFERENCES

Abrahams, M.R., Anderson, J.A., Giorgi, E.E., Seoighe, C., Mlisana, K., Ping, L.H., Athreya, G.S., Treurnicht, F.K., Keele, B.F., Wood, N., et al.; CAPRISA Acute Infection Study Team; Center for HIV-AIDS Vaccine Immunology Consortium (2009). Quantitating the multiplicity of infection with human immunodeficiency virus type 1 subtype C reveals a non-poisson distribution of transmitted variants. J. Virol. *83*, 3556–3567.

Ackerman, M.E., Mikhailova, A., Brown, E.P., Dowell, K.G., Walker, B.D., Bailey-Kellogg, C., Suscovich, T.J., and Alter, G. (2016). Polyfunctional HIV-specific antibody responses are associated with spontaneous HIV control. PLoS Pathog. *12*, e1005315.

Alpert, M.D., Heyer, L.N., Williams, D.E.J., Harvey, J.D., Greenough, T., Allhorn, M., and Evans, D.T. (2012). A novel assay for antibody-dependent cell-mediated cytotoxicity against HIV-1- or SIV-infected cells reveals incomplete overlap with antibodies measured by neutralization and binding assays. J. Virol. *86*, 12039–12052.

Balasubramanian, P., Williams, C., Shapiro, M.B., Sinangil, F., Higgins, K., Nádas, A., Totrov, M., Kong, X.-P., Fiore-Gartland, A.J., Haigwood, N.L., et al. (2018). Functional antibody response against V1V2 and V3 of HIV gp120 in the VAX003 and VAX004 vaccine trials. Sci. Rep. *8*, 542.

Bar, K.J., Li, H., Chamberland, A., Tremblay, C., Routy, J.P., Grayson, T., Sun, C., Wang, S., Learn, G.H., Morgan, C.J., et al. (2010). Wide variation in the multiplicity of HIV-1 infection among injection drug users. J. Virol. *84*, 6241–6247.

Barnett, S.W., Srivastava, I.K., Kan, E., Zhou, F., Goodsell, A., Cristillo, A.D., Ferrai, M.G., Weiss, D.E., Letvin, N.L., Montefiori, D., et al. (2008). Protection of macaques against vaginal SHIV challenge by systemic or mucosal and systemic vaccinations with HIV-envelope. AIDS *22*, 339–348.

Barnett, S.W., Burke, B., Sun, Y., Kan, E., Legg, H., Lian, Y., Bost, K., Zhou, F., Goodsell, A., Zur Megede, J., et al. (2010). Antibody-mediated protection against mucosal simian-human immunodeficiency virus challenge of macaques immunized with alphavirus replicon particles and boosted with trimeric envelope glycoprotein in MF59 adjuvant. J. Virol. *84*, 5975–5985.

Barouch, D.H., Alter, G., Broge, T., Linde, C., Ackerman, M.E., Brown, E.P., Borducchi, E.N., Smith, K.M., Nkolola, J.P., Liu, J., et al. (2015). Protective efficacy of adenovirus/protein vaccines against SIV challenges in rhesus monkeys. Science *349*, 320–324.

Bianchi, M., Turner, H.L., Nogal, B., Cottrell, C.A., Oyen, D., Pauthner, M., Bastidas, R., Nedellec, R., McCoy, L.E., Wilson, I.A., et al. (2018). Electron-microscopy-based epitope mapping defines specificities of polyclonal antibodies elicited during HIV-1 BG505 envelope trimer immunization. Immunity *49*, 288–300.e8.

Bukh, I., Calcedo, R., Roy, S., Carnathan, D.G., Grant, R., Qin, Q., Boyd, S., Ratcliffe, S.J., Veeder, C.L., Bellamy, S.L., et al. (2014). Increased mucosal CD4+ T cell activation in rhesus macaques following vaccination with an adenoviral vector. J. Virol. *88*, 8468–8478.

Carnathan, D.G., Wetzel, K.S., Yu, J., Lee, S.T., Johnson, B.A., Paiardini, M., Yan, J., Morrow, M.P., Sardesai, N.Y., Weiner, D.B., et al. (2015). Activated CD4+CCR5+ T cells in the rectum predict increased SIV acquisition in SIVGag/Tat-vaccinated rhesus macaques. Proc. Natl. Acad. Sci. USA *112*, 518–523.

Cline, A.N., Bess, J.W., Piatak, M., Jr., and Lifson, J.D. (2005). Highly sensitive SIV plasma viral load assay: practical considerations, realistic performance expectations, and application to reverse engineering of vaccines for AIDS. J. Med. Primatol. *34*, 303–312.

Cohen, Y.Z., Lorenzi, J.C.C., Seaman, M.S., Nogueira, L., Schoofs, T., Krassnig, L., Butler, A., Millard, K., Fitzsimons, T., Daniell, X., et al. (2018). Neutralizing activity of broadly neutralizing anti-HIV-1 antibodies against clade B clinical isolates produced in peripheral blood mononuclear cells. J. Virol. *92*, 92.

de Taeye, S.W., Ozorowski, G., Torrents de la Peña, A., Guttman, M., Julien, J.-P., van den Kerkhof, T.L.G.M., Burger, J.A., Pritchard, L.K., Pugach, P., Yasmeen, A., et al. (2015). Immunogenicity of stabilized HIV-1 envelope trimers with reduced exposure of non-neutralizing epitopes. Cell *163*, 1702–1715.

Del Prete, G.Q., Keele, B.F., Fode, J., Thummar, K., Swanstrom, A.E., Rodriguez, A., Raymond, A., Estes, J.D., LaBranche, C.C., Montefiori, D.C., et al. (2017). A single gp120 residue can affect HIV-1 tropism in macaques. PLoS Pathog. *13*, e1006572.

Ding, S., Veillette, M., Coutu, M., Prévost, J., Scharf, L., Bjorkman, P.J., Ferrari, G., Robinson, J.E., Stürzel, C., Hahn, B.H., et al. (2015). A highly conserved residue of the HIV-1 gp120 inner domain is important for antibody-dependent cellular cytotoxicity responses mediated by anti-cluster A antibodies. J. Virol. *90*, 2127–2134.

Eren, K., Weaver, S., Ketteringham, R., Valentyn, M., Laird Smith, M., Kumar, V., Mohan, S., Kosakovsky-Pond, S.L., and Murrell, B. (2017). Full-Length Envelope Analyzer (FLEA): A tool for longitudinal analysis of viral amplicons. bioRxiv. https://doi.org/10.1101/230474.

Exler, J.-L., Ake, J., Robb, M.L., Kim, J.H., and Plotkin, S.A. (2014). Nonneutralizing functional antibodies: a new "old" paradigm for HIV vaccines. Clin. Vaccine Immunol. *21*, 1023–1036.

Fauci, A.S., Marovich, M.A., Dieffenbach, C.W., Hunter, E., and Buchbinder, S.P. (2014). Immunology. Immune activation with HIV vaccines. Science *344*, 49–51.

Fenizia, C., Keele, B.F., Nichols, D., Cornara, S., Binello, N., Vaccari, M., Pegu, P., Robert-Guroff, M., Ma, Z.-M., Miller, C.J., et al. (2011). TRIM5α does not affect simian immunodeficiency virus SIV(mac251) replication in vaccinated or unvaccinated Indian rhesus macaques following intrarectal challenge exposure. J. Virol. *85*, 12399–12409.

Ferrari, G., Pollara, J., Kozink, D., Harms, T., Drinker, M., Freel, S., Moody, M.A., Alam, S.M., Tomaras, G.D., Ochsenbauer, C., et al. (2011). An HIV-1 gp120 envelope human monoclonal antibody that recognizes a C1 conformational epitope mediates potent antibody-dependent cellular cytotoxicity (ADCC) activity and defines a common ADCC epitope in human HIV-1 serum. J. Virol. *85*, 7029–7036.

Fouts, T.R., Bagley, K., Prado, I.J., Bobb, K.L., Schwartz, J.A., Xu, R., Zagursky, R.J., Egan, M.A., Eldridge, J.H., LaBranche, C.C., et al. (2015). Balance of cellular and humoral immunity determines the level of protection by HIV vaccines in rhesus macaque models of HIV infection. Proc. Natl. Acad. Sci. USA *112*, E992–E999.

Havenar-Daughton, C., Carnathan, D.G., Torrents de la Peña, A., Pauthner, M., Briney, B., Reiss, S.M., Wood, J.S., Kaushik, K., van Gils, M.J., Rosales, S.L.,

et al. (2016). Direct probing of germinal center responses reveals immunological features and bottlenecks for neutralizing antibody responses to HIV Env trimer. Cell Rep. *17*, 2195–2209.

Havenar-Daughton, C., Lee, J.H., and Crotty, S. (2017). Tfh cells and HIV bnAbs, an immunodominance model of the HIV neutralizing antibody generation problem. Immunol. Rev. *275*, 49–61.

Haynes, B.F., and Burton, D.R. (2017). Developing an HIV vaccine. Science *355*, 1129–1130.

Haynes, B.F., Gilbert, P.B., McElrath, M.J., Zolla-Pazner, S., Tomaras, G.D., Alam, S.M., Evans, D.T., Montefiori, D.C., Karnasuta, C., Sutthent, R., et al. (2012). Immune-correlates analysis of an HIV-1 vaccine efficacy trial. N. Engl. J. Med. *366*, 1275–1286.

Hessell, A.J., Malherbe, D.C., and Haigwood, N.L. (2018). Passive and active antibody studies in primates to inform HIV vaccines. Expert Rev. Vaccines *17*, 127–144.

Hu, H., Eller, M.A., Zafar, S., Zhou, Y., Gu, M., Wei, Z., Currier, J.R., Marovich, M.A., Kibuuka, H.N., Bailer, R.T., et al. (2014). Preferential infection of human Ad5-specific CD4 T cells by HIV in Ad5 naturally exposed and recombinant Ad5-HIV vaccinated individuals. Proc. Natl. Acad. Sci. USA *111*, 13439–13444.

Huang, Y., Ferrari, G., Alter, G., Forthal, D.N., Kappes, J.C., Lewis, G.K., Love, J.C., Borate, B., Harris, L., Greene, K., et al. (2016). Diversity of antiviral IgG effector activities observed in HIV-infected and vaccinated subjects. J. Immunol. *197*, 4603–4612.

Johansson, S.E., Rollman, E., Chung, A.W., Center, R.J., Hejdeman, B., Stratov, I., Hinkula, J., Wahren, B., Kärre, K., Kent, S.J., and Berg, L. (2011). NK cell function and antibodies mediating ADCC in HIV-1-infected viremic and controller patients. Viral Immunol. *24*, 359–368.

Keele, B.F., Li, W., Borducchi, E.N., Nkolola, J.P., Abbink, P., Chen, B., Seaman, M.S., and Barouch, D.H. (2017). Adenovirus prime, Env protein boost vaccine protects against neutralization-resistant SIVsmE660 variants in rhesus monkeys. Nat. Commun. *8*, 15740.

Kirmaier, A., Wu, F., Newman, R.M., Hall, L.R., Morgan, J.S., O'Connor, S., Marx, P.A., Meythaler, M., Goldstein, S., Buckler-White, A., et al. (2010). TRIM5 suppresses cross-species transmission of a primate immunodeficiency virus and selects for emergence of resistant variants in the new species. PLoS Biol. *8*, 8.

Klasse, P.J., Ketas, T.J., Cottrell, C.A., Ozorowski, G., Debnath, G., Camara, D., Francomano, E., Pugach, P., Ringe, R.P., LaBranche, C.C., et al. (2018). Epitopes for neutralizing antibodies induced by HIV-1 envelope glycoprotein BG505 SOSIP trimers in rabbits and macaques. PLoS Pathog. *14*, e1006913.

Krammer, F., and Palese, P. (2015). Advances in the development of influenza virus vaccines. Nat. Rev. Drug Discov. *14*, 167–182.

Kristensen, A.B., Kent, S.J., and Parsons, M.S. (2018). Contribution of NK cell education to both direct and anti-HIV-1 antibody-dependent NK cell functions. J. Virol. *92*, e02146-17.

Kulp, D.W., Steichen, J.M., Pauthner, M., Hu, X., Schiffner, T., Liguori, A., Cottrell, C.A., Havenar-Daughton, C., Ozorowski, G., Georgeson, E., et al. (2017). Structure-based design of native-like HIV-1 envelope trimers to silence non-neutralizing epitopes and eliminate CD4 binding. Nat. Commun. *8*, 1655.

Laird Smith, M., Murrell, B., Eren, K., Ignacio, C., Landais, E., Weaver, S., Phung, P., Ludka, C., Hepler, L., Caballero, G., et al. (2016). Rapid sequencing of complete env genes from primary HIV-1 samples. Virus. Evol *2*, vew018.

Li, H., Bar, K.J., Wang, S., Decker, J.M., Chen, Y., Sun, C., Salazar-Gonzalez, J.F., Salazar, M.G., Learn, G.H., Morgan, C.J., et al. (2010). High multiplicity infection by HIV-1 in men who have sex with men. PLoS Pathog. *6*, e1000890.

Li, H., Wang, S., Kong, R., Ding, W., Lee, F.-H., Parker, Z., Kim, E., Learn, G.H., Hahn, P., Policicchio, B., et al. (2016). Envelope residue 375 substitutions in simian-human immunodeficiency viruses enhance CD4 binding and replication in rhesus macaques. Proc. Natl. Acad. Sci. USA *113*, E3413–E3422.

Liu, J., Keele, B.F., Li, H., Keating, S., Norris, P.J., Carville, A., Mansfield, K.G., Tomaras, G.D., Haynes, B.F., Kolodkin-Gal, D., et al. (2010). Low-dose mucosal simian immunodeficiency virus infection restricts early replication kinetics and transmitted virus variants in rhesus monkeys. J. Virol. *84*, 10406–10412.

Martins, M.A., and Watkins, D.I. (2018). What is the predictive value of animal models for vaccine efficacy in humans? Rigorous simian immunodeficiency virus vaccine trials can be instructive. Cold Spring Harb. Perspect. Biol. *10*, a029504.

McCoy, L.E., van Gils, M.J., Ozorowski, G., Messmer, T., Briney, B., Voss, J.E., Kulp, D.W., Macauley, M.S., Sok, D., Pauthner, M., et al. (2016). Holes in the glycan shield of the native HIV envelope are a target of trimer-elicited neutralizing antibodies. Cell Rep. *16*, 2327–2338.

Moldt, B., Rakasz, E.G., Schultz, N., Chan-Hui, P.-Y., Swiderek, K., Weisgrau, K.L., Piaskowski, S.M., Bergman, Z., Watkins, D.I., Poignard, P., and Burton, D.R. (2012). Highly potent HIV-specific antibody neutralization in vitro translates into effective protection against mucosal SHIV challenge in vivo. Proc. Natl. Acad. Sci. USA *109*, 18921–18925.

Pal, R., Kalyanaraman, V.S., Nair, B.C., Whitney, S., Keen, T., Hocker, L., Hudacik, L., Rose, N., Mboudjeka, I., Shen, S., et al. (2006). Immunization of rhesus macaques with a polyvalent DNA prime/protein boost human immunodeficiency virus type 1 vaccine elicits protective antibody response against simian human immunodeficiency virus of R5 phenotype. Virology *348*, 341–353.

Parren, P.W., Marx, P.A., Hessell, A.J., Luckay, A., Harouse, J., Cheng-Mayer, C., Moore, J.P., and Burton, D.R. (2001). Antibody protects macaques against vaginal challenge with a pathogenic R5 simian/human immunodeficiency virus at serum levels giving complete neutralization in vitro. J. Virol. *75*, 8340–8347.

Pauthner, M., Havenar-Daughton, C., Sok, D., Nkolola, J.P., Bastidas, R., Boopathy, A.V., Carnathan, D.G., Chandrashekar, A., Cirelli, K.M., Cottrell, C.A., et al. (2017). Elicitation of robust tier 2 neutralizing antibody responses in nonhuman primates by HIV envelope trimer immunization using optimized approaches. Immunity *46*, 1073–1088.e6.

Pegu, A., Yang, Z.-Y., Boyington, J.C., Wu, L., Ko, S.-Y., Schmidt, S.D., McKee, K., Kong, W.-P., Shi, W., Chen, X., et al. (2014). Neutralizing antibodies to HIV-1 envelope protect more effectively in vivo than those to the CD4 receptor. Sci. Transl. Med. *6*, 243ra88.

Provine, N.M., Cortez, V., Chohan, V., and Overbaugh, J. (2012). The neutralization sensitivity of viruses representing human immunodeficiency virus type 1 variants of diverse subtypes from early in infection is dependent on producer cell, as well as characteristics of the specific antibody and envelope variant. Virology *427*, 25–33.

Qureshi, H., Ma, Z.-M., Huang, Y., Hodge, G., Thomas, M.A., DiPasquale, J., DeSilva, V., Fritts, L., Bett, A.J., Casimiro, D.R., et al. (2012). Low-dose penile SIVmac251 exposure of rhesus macaques infected with adenovirus type 5 (Ad5) and then immunized with a replication-defective Ad5-based SIV gag/pol/nef vaccine recapitulates the results of the phase IIb step trial of a similar HIV-1 vaccine. J. Virol. *86*, 2239–2250.

Reiss, S., Baxter, A.E., Cirelli, K.M., Dan, J.M., Morou, A., Daigneault, A., Brassard, N., Silvestri, G., Routy, J.P., Havenar-Daughton, C., et al. (2017). Comparative analysis of activation induced marker (AIM) assays for sensitive identification of antigen-specific CD4 T cells. PLoS ONE *12*, e0186998.

Richard, J., Prévost, J., Baxter, A.E., von Bredow, B., Ding, S., Medjahed, H., Delgado, G.G., Brassard, N., Stürzel, C.M., Kirchhoff, F., et al. (2018). Uninfected bystander cells impact the measurement of HIV-specific antibody-dependent cellular cytotoxicity responses. MBio *9*, e00358.

Roederer, M., Keele, B.F., Schmidt, S.D., Mason, R.D., Welles, H.C., Fischer, W., Labranche, C., Foulds, K.E., Louder, M.K., Yang, Z.-Y., et al. (2014). Immunological and virological mechanisms of vaccine-mediated protection against SIV and HIV. Nature *505*, 502–508.

Sanders, R.W., Derking, R., Cupo, A., Julien, J.-P., Yasmeen, A., de Val, N., Kim, H.J., Blattner, C., de la Peña, A.T., Korzun, J., et al. (2013). A next-generation cleaved, soluble HIV-1 Env trimer, BG505 SOSIP.664 gp140, expresses multiple epitopes for broadly neutralizing but not non-neutralizing antibodies. PLoS Pathog. *9*, e1003618–e1003618.

Sanders, R.W., van Gils, M.J., Derking, R., Sok, D., Ketas, T.J., Burger, J.A., Ozorowski, G., Cupo, A., Simonich, C., Goo, L., et al. (2015). HIV-1

VACCINES. HIV-1 neutralizing antibodies induced by native-like envelope trimers. Science *349*, aac4223–aac4223.

Shingai, M., Donau, O.K., Plishka, R.J., Buckler-White, A., Mascola, J.R., Nabel, G.J., Nason, M.C., Montefiori, D., Moldt, B., Poignard, P., et al. (2014). Passive transfer of modest titers of potent and broadly neutralizing anti-HIV monoclonal antibodies block SHIV infection in macaques. J. Exp. Med. *211*, 2061–2074.

Sok, D., Le, K.M., Vadnais, M., Saye-Francisco, K.L., Jardine, J.G., Torres, J.L., Berndsen, Z.T., Kong, L., Stanfield, R., Ruiz, J., et al. (2017). Rapid elicitation of broadly neutralizing antibodies to HIV by immunization in cows. Nature *548*, 108–111.

Staprans, S.I., Barry, A.P., Silvestri, G., Safrit, J.T., Kozyr, N., Sumpter, B., Nguyen, H., McClure, H., Montefiori, D., Cohen, J.I., and Feinberg, M.B. (2004). Enhanced SIV replication and accelerated progression to AIDS in macaques primed to mount a CD4 T cell response to the SIV envelope protein. Proc. Natl. Acad. Sci. USA *101*, 13026–13031.

Stephenson, K.E., D’Couto, H.T., and Barouch, D.H. (2016). New concepts in HIV-1 vaccine development. Curr. Opin. Immunol. *41*, 39–46.

Sundling, C., Martinez, P., Soldemo, M., Spångberg, M., Bengtsson, K.L., Stertman, L., Forsell, M.N.E., and Karlsson Hedestam, G.B. (2013). Immunization of macaques with soluble HIV type 1 and influenza virus envelope glycoproteins results in a similarly rapid contraction of peripheral B-cell responses after boosting. J. Infect. Dis. *207*, 426–431.

Tomaras, G.D., and Plotkin, S.A. (2017). Complex immune correlates of protection in HIV-1 vaccine efficacy trials. Immunol. Rev. *275*, 245–261.

Torrents de la Peña, A., Julien, J.-P., de Taeye, S.W., Garces, F., Guttman, M., Ozorowski, G., Pritchard, L.K., Behrens, A.-J., Go, E.P., Burger, J.A., et al. (2017). Improving the immunogenicity of native-like HIV-1 envelope trimers by hyperstabilization. Cell Rep. *20*, 1805–1817.

Torrents de la Peña, A., de Taeye, S.W., Sliepen, K., LaBranche, C.C., Burger, J.A., Schermer, E.E., Montefiori, D.C., Moore, J.P., Klasse, P.J., and Sanders, R.W. (2018). Immunogenicity in rabbits of HIV-1 SOSIP trimers from clades A, B and C, given individually, sequentially or in combinations. J. Virol. *92*, 92.

Veillette, M., Coutu, M., Richard, J., Batraville, L.-A., Désormeaux, A., Roger, M., and Finzi, A. (2014). Conformational evaluation of HIV-1 trimeric envelope glycoproteins using a cell-based ELISA assay. J. Vis. Exp. *91*, 51995.

Veillette, M., Coutu, M., Richard, J., Batraville, L.-A., Dagher, O., Bernard, N., Tremblay, C., Kaufmann, D.E., Roger, M., and Finzi, A. (2015). The HIV-1 gp120 CD4-bound conformation is preferentially targeted by antibody-dependent cellular cytotoxicity-mediating antibodies in sera from HIV-1-infected individuals. J. Virol. *89*, 545–551.

von Bredow, B., Arias, J.F., Heyer, L.N., Moldt, B., Le, K., Robinson, J.E., Zolla-Pazner, S., Burton, D.R., and Evans, D.T. (2016). Comparison of antibody-dependent cell-mediated cytotoxicity and virus neutralization by HIV-1 Env-specific monoclonal antibodies. J. Virol. *90*, 6127–6139.

STAR★METHODS

KEY RESOURCES TABLE

REAGENT or RESOURCE	SOURCE	IDENTIFIER
Antibodies		
CD4 (Clone SK3) PerCP eFluor710	Invitrogen	Cat# 46-0047-42
CD20 (Clone 2H7) BV570	BioLegend	Cat# 302332
CD8 (Clone RPA-T8) BV650	BD Biosciences	Cat# 563821
CCR5 (3A9) APC	BD Biosciences	Cat# 550856
α4β7 (Act-1) PE	NHP Reagent Resource	Cat# Alpha-4/beta-7-PE
Live/Dead APC e780	Invitrogen	Cat# 65086518
Ki67 (B56) Ax488	BD Biosciences	Cat# 558616
IL2 (MQ1-17H12) Ax700	BioLegend	Cat# 500320
IFNγ (Clone B27) Pac Blue	Invitrogen	Cat# 50-113-7517
TNF (MAb11) PECy7	BD Biosciences	Cat# 557647
CD40L (24-31) BV605	BioLegend	Cat# 310826
CD4 (Clone OKT-4) BV650	BioLegend	Cat# 317436
CD20 (Clone 2H7) BV570	BioLegend	Cat# 302332
PD1 (Clone EH12.2H7) BV785	BioLegend	Cat# 329930
CXCR5 (Clone MU5UBEE) PECy7	Invitrogen	Cat# 25-9185-41
CD14 (61D3) APC e780	Invitrogen	Cat# 47-0149-42
CD16 (eBioCD16) APC e780	Invitrogen	Cat# 47-0168-42
CD25 (Clone BC96) FITC	BioLegend	Cat# 302604
Ox40 (Clone L106) PE	BD Biosciences	Cat# 340420
4-1-BB (4B4-1) BV421	BD Biosciences	Cat# 564091
ICOS (C398.4A) PerCP Cy5.5	BioLegend	Cat# 313518
CXCR3 (1C6) APC	BD Biosciences	Cat# 561732
AquaVivid	ThermoFisher Scientific	Cat# L34957
Cell proliferation dye eFluor670	ThermoFisher Scientific	Cat# 65-0840-85
Cell proliferation dye eFluor450	ThermoFisher Scientific	Cat# 65-0842-85
Anti-SIV$_{mac}$ p27 Hybridoma (clone 2F12)	NIH AIDS Reagent Program	Cat# 1547
Alexa Fluor 488 Protein Labeling Kit	ThermoFisher Scientific	Cat# A10235
Bacterial and Virus Strains		
BG505.W6M.ENV.C2	NIH AIDS Reagent Program	Cat# 11518
rhCD4$^+$ T-cell-grown SHIV$_{BG505}$ N332 S375Y challenge stock	Li et al., 2016	N/A
Chemicals, Peptides, and Recombinant Proteins		
BG505 SOSIP.664	Sanders et al., 2013	N/A
BG505 SOSIP.v4.1	de Taeye et al., 2015	N/A
BG505 SOSIP.v5.2	Torrents de la Peña et al., 2017	N/A
BG505 Olio6	Kulp et al., 2017	N/A
BG505 Olio6 CD4-KO	Kulp et al., 2017	N/A
BG505 V3-peptide TRPNNNTRKSIRIGPGQAFYATG	A&A Labs LLC	N/A
Cholesterol	Avanti Polar Lipids	Cat# 700000
DPPC (1,2-dipalmitoyl-sn-glycero-3-phosphocholine)	Avanti Polar Lipids	Cat# 850355
Quil-A	InvivoGen	Cat# vac-quil
MEGA-10	Sigma	Cat# D62777
Critical Commercial Assays		
Limulus Amebocyte Lysate assay	Lonza	Cat# QCL-1000
Cholesterol quantification kit	Sigma	Cat# MAK043

(*Continued on next page*)

Continued		
REAGENT or RESOURCE	SOURCE	IDENTIFIER
Experimental Models: Cell Lines		
TZM-bl cells	NIH AIDS Reagent Program	Cat# 8129
CEM.NKR-CCR5-sLTR-Luc cells	Alpert et al., 2012	N/A
CD16$^+$ NK effector cell line	Alpert et al., 2012	N/A
Experimental Models: Organisms/Strains		
Indian-origin rhesus macaques (outbred)	AlphaGenesis Inc	N/A
Oligonucleotides		
Env-F: GAGCAGAAGACAGTGGCAATGA	IDT	N/A
SHIV-R: CCCTGATTGTATTTCTGTCCCTCAC	IDT	N/A
sGag21 GTCTGCGTCATCTGGTGCATTC (fwd)	N/A	N/A
sGag22 CACTAGGTGTCTCTGCACTATCTGTTTTG (rev)	N/A	N/A
sGag23 CTTCCTCAGTGTGTTTCACTTTCTCTTCTGCG (probe)	N/A	N/A
Recombinant DNA		
pSG3Δenv plasmid	NIH AIDS Reagent Program	Cat# 11051
Software and Algorithms		
Prism v7.0	Graphpad	https://www.graphpad.com/scientific-software/prism/
FlowJo v10.0.7	FlowJo LLC	https://www.flowjo.com
Full-Length Env Analyzer (FLEA)	Eren et al., 2017; Laird Smith et al., 2016	http://datamonkey.org/flea

CONTACT FOR REAGENT AND RESOURCE SHARING

Further information and requests for resources and reagents should be directed to and will be fulfilled by the Lead Contact, Dennis Burton (burton@scripps.edu).

EXPERIMENTAL MODEL AND SUBJECT DETAILS

Outbred Indian rhesus macaques (*Macaca mulatta*) were sourced and housed at Alphagenesis Inc, Yemasee, SC and maintained in accordance with NIH guidelines. These studies were approved by the appropriate Institutional Animal Care and Use Committees (IACUC). None of the NHPs were previously enrolled in other studies that are not explicity stated in the manuscript. All animals were genotyped for class I alleles Mamu-A*01, Mamu-B*08 and Mamu-B*17 and Trim5, which are associated with spontaneous virological control. Genotype and gender information for all animals is reported in Table S1. Additional information on high and low nAb titer group animals is published in Pauthner et al. (2017).

METHOD DETAILS

Rhesus monkey immunizations and challenge

Animals were immunized at 4 weeks before challenge (week −4) with this fourth immunization for a given animal being identical with that previously administered to that animal (Figure S1B) (Pauthner et al., 2017). The adjuvant used for this boost was a soluble ISCOMs-class saponin comprised of self-assembled cholesterol, phospholipid, and Quillaja saponin. Briefly, 10 mg each of cholesterol (Avanti Polar Lipids 700000) and DPPC (Avanti Polar Lipids 850355) were dissolved separately in 20% MEGA-10 (Sigma D6277) detergent at a final concentration of 20 mg/ml and 50 mg Quil-A saponin (InvivoGen vac-quil) was dissolved in MilliQ H_2O at a final concentration of 100 mg/ml. Next, DPPC solution was added to cholesterol followed by addition of Quil-A saponin in rapid succession and the volume was brought up with PBS for a final concentration of 1 mg/ml cholesterol and 2% MEGA-10. The solution was allowed to equilibrate at 25°C overnight, followed by 5 days of dialysis against PBS using a 10k MWCO membrane. The adjuvant solution was then filter sterilized using a 0.2 μm Supor syringe filter, and concentrated using 50k MWCO centricon filters. Each adjuvant batch was finally characterized by negative stain transmission electron microscopy (TEM) and dynamic light scattering (DLS) to confirm uniform morphology and size. The adjuvant was also validated for low endotoxin contect by Limulus Amebocyte Lystae assay (Lonza QCL-1000). Final adjuvant concentration was determined by cholesterol quantification (Sigma MAK043).

All immunizations were administered as split doses. Each immunization consisted of two subcutaneous injections of 50 μg of Env trimer protein + 187.5 units (U) of saponin adjuvant, in sterile phosphate-buffered saline (PBS) diluent for a total of 100 μg of Env trimer

protein + 375 U of a soluble ISCOMs-class saponin adjuvant per immunization per animal. Units of the ISCOMs-like adjuvant were defined as theoretical micrograms of Quil A in the dose, determined from direct measurements of cholesterol incorporation and inferring Quil A content from the input 5:1 Quil A:cholesterol ratio. Subcutaneous immunizations were given in a volume of 0.5 mL with a 1 inch, 25-gauge needle at the medial inner mid-thigh of each leg. The subcutaneous injection technique consists of making a 'skin tent' and inserting the needle into the subcutaneous space at a 45 angle.

Serum was collected in SST Vaccutainer tubes (BD Biosciences) and processed according to the manufacturer's instructions. Multiple aliquots of 0.5 mL were frozen at -80°C. Whole blood was collected in K2 EDTA Vaccutainer tubes (BD Biosciences) for plasma and PBMC isolation. Multiple aliquots of 0.5 mL of plasma were frozen at -80°C. PBMCs were isolated using Thermo Scientific Nunc EZFlip Conical Centrifuge Tubes per manufacturer's instructions. PBMCs were isolated, counted, and re-suspended at 1×10^7 cells/mL in FBS containing 10% DMSO. Aliquots were subsequently frozen in 1 mL vials using a Mr. Frosty freezing container (Nalgene, cooling rate of 1°C / minute) and placed in a -80°C freezer. The following day PBMC samples were moved to storage in a liquid nitrogen freezer tank.

Animals were atraumatically inoculated intrarectally with a 1:75 dilution of rhCD4$^+$ T-cell-grown SHIV$_{BG505}$ N332 S375Y ΔCT challenge stock (Li et al., 2016) in RPMI 1640 (GIBCO), which amounted to 1.4 x10^7 virions or 2 ng p27. See dataset S1B in Li et al. (Li et al., 2016) for a complete characterization of the challenge stock with respect to virion content and virion infectivity.

Viral Load Assay

RT-PCR assays were utilized to monitor viral loads, essentially as previously described (Cline et al., 2005). RNA was extracted from plasma with a QIAcube HT (QIAGEN, Germany) using the Qiacube 96 Cador pathogen HT. The SIV *gag* gene was utilized as a standard. RNA standards were generated using the AmpliCap-Max T7 High Yield Message Maker Kit (Cell Script) and purified with RNA clean and concentrator kit (Zymo Research, CA, USA). RNA quality and concentration was assessed by the BIDMC Molecular Core Facility. Log dilutions of the RNA standard were included with each RT-PCR assay. Reverse transcription of both standards and samples was done using Superscript III VILO (Invitrogen). RT-PCRs with primers sGag21, sGag22 and probe sGag23 were run on the Quantstudio 6 Flex system (Applied Biosystems). Viral loads were calculated as virus particles (VP) per ml. Assay sensitivity was > 100 copies/ml.

Primers used for RT-PCR Reactions:

sGag21 GTCTGCGTCATCTGGTGCATTC (fwd)
sGag22 CACTAGGTGTCTCTGCACTATCTGTTTTG (rev)
sGag23 CTTCCTCAGTGTGTTTCACTTTCTCTTCTGCG (probe)

Serum neutralization assays

Replication incompetent HIV pseudovirus was produced by co-transfecting *env* plasmids with an *env*-deficient backbone plasmid (pSG3Δ*env*) in HEK293T cells in a 1:2 ratio, using the X-tremeGENE 9 transfection reagent (Roche). Pseudovirus was harvested after 48-72 h by sterile-filtration (0.22 μm) of cell culture supernatants, and neutralization was tested by incubating pseudovirus and serum or mAbs for 1 h at 37°C before transferring them onto TZM-bl cells as previously described (Pauthner et al., 2017). In brief, supernatant was removed from TZM-bl cells after 48-72 h, cells were then lysed, and luciferase activity was measured following addition of Bright-Glo (Promega) according to manufacturer's instructions. For replication competent SHIV$_{BG505}$ neutralization, rhCD4$^+$ T-cell-grown SHIV$_{BG505}$ N332 S375Y challenge stock was used instead in a BSL3 facility with no further modifications.

Neutralization is measured in duplicate wells within each experiment. BG505 nAb titers for group comparisons were measured in three or more independent experiments that were subsequently averaged. The BG505 pseudovirus time course neutralization data shown in Figure 3 were generated in single large experiments, to test sera from all time points side-by-side, thus ensuring the highest nAb titer comparability between time points. Neutralization was tested starting at 1:10 serum dilutions followed by nine serial 3-fold dilutions to ensure the highest sensitivity and range of detection. Neutralization ID$_{50}$ titers were calculated using the 'One site – Fit logIC$_{50}$' regression in Graphpad Prism v7.0. ID$_{50}$ nAb titers of incomplete neutralization curves that reached at least 50%, but less than 90% maximal neutralization, were calculated by constraining the regression fit through 0% and 100% neutralization, to ensure accurate calculation of half-way (50%) nAb titers. All neutralization titers are reported as ID$_{50}$ titers. All nAb titer data panels show geometric mean titers with geometric SD. BG505 pseudovirus neutralization was tested using the BG505.W6M.ENV.C2 isolate (AIDS Reagents Program), carrying the T332N mutation to restore the N332 glycosylation site, as well as other indicated mutations that were added by site-directed mutagenesis.

Serum binding ELISAs

Microlon 96-well plates (Corning) were coated overnight with streptavidin at 2.5 μg/mL (Thermo Scientific). Plates were then washed 4-5 times with PBS-tween (0.05%) and blocked with PBS + 3% BSA for 1 h at room temperature. If capturing biotinylated BG505 SOSIP.664-Avi or BG505-Avi gp120, proteins were added at 2.5 μg/mL in PBS + 1% BSA for 2 h at room temperature. For V3-peptide binding assays, no streptavidin was coated and instead BG505 V3-peptide (TRPNNNTRKSIRIGPGQAFYATG) was directly coated to Microlon 96-well plates at 2.5 μg/mL in PBS overnight. Plates were then washed 4-5 times with PBS-tween (0.05%) and serially diluted sera in PBS + 1% BSA were then added for 1 h at room temperature. Plates were then washed 4-5 times with PBS-tween

(0.05%) and alkaline phosphatase-conjugated goat anti-human IgG (Jackson ImmunoResearch) was added for 1 h at a 1:1000 dilution (final concentration 0.33 μg/mL) in PBS + 1% BSA at room temperature. Plates were then washed 4-5 times with PBS-tween (0.05%) and absorption at 405 nm was measured following addition of phosphatase substrate in alkaline phosphatase buffer. We calculated half maximal EC_{50} binding titers using Graphpad Prism v7.0. All ELISA Ab data panels show geometric mean titers with geometric SD.

ADCC assays

Luciferase-based CEM.NKR SHIV, HIV, SIV infection assay

ADCC activity was measured as previously described (Alpert et al., 2012). CEM.NKR-$_{CCR5}$-sLTR-Luc cells, which express luciferase (Luc) upon infection, were infected with either HIV-1 BG505, SHIV BG505 or SIV_{mac}239 by spinoculation in the presence of 40 μg/ml of polybrene. For HIV-1 BG505 and $SHIV_{BG505}$ infections, *vif*-deleted infectious molecular clones were pseudotyped with Vesicular stomatitis virus G (VSVG). Two days post-infection with VSVG-pseudotyped HIV-1/$SHIV_{BG505}$ and 4 days post-infection with SIV-$_{mac}$239, CEM.NKR-$_{CCR5}$-sLTR-Luc cells were incubated at a 10:1 effector:target cell ratio either with an NK cell line expressing rhesus macaque CD16 in the presence of serial dilutions of rhesus macaque sera or an NK cell line expressing human CD16 in the presence of human monoclonal bnAbs. After an 8-hour incubation, Luc activity was measured using BriteLite luciferase substrate (PerkinElmer). Uninfected or infected cells incubated with NK cells in the absence of antibody or plasma were used to determine background and maximal Luc activity, respectively. The dose-dependent loss of Luc activity represents the antibody-dependent killing of productively infected target cells.

FACS-based CEM.NKR SHIV infection assay

VSVG-pseudotyped $SHIV_{BG505}$ N332 S375Y virus was produced and titrated as previously described (Veillette et al., 2015). Viruses were then used to infect CEM.NKR-CCR5-sLTR-Luc cells by spin infection at 800 × *g* for 1 h in 96-well plates at 25°C. Measurement of ADCC using the FACS-based assay was performed at 48h post-infection as previously described (Veillette et al., 2015). Briefly, infected CEM.NKR-CCR5-sLTR-Luc cells were stained with viability (AquaVivid; Thermo Fisher Scientific) and cellular (cell proliferation dye eFluor670; eBioscience) markers and used as target cells. Human PBMCs isolated from three different healthy HIV-uninfected individuals were used as effector cells and were stained with another cellular marker (cell proliferation dye eFluor450; eBioscience). Effector cells were added at an effector:target cell ratio of 10:1 in 96-well V-bottom plates (Corning, Corning, NY). A 1:250 final dilution of sera or 5 μg/ml of mAbs were added to appropriate wells and cells were incubated for 15 min at room temperature. The plates were subsequently centrifuged for 1 min at 300 x g, and incubated at 37°C, 5% CO_2 for 5 to 6 h before being fixed with a PBS-formaldehyde solution (2% formaldehyde final concentration). Cells were then permeabilized using the Cytofix/Cytoperm Fixation/Permeabilization Kit (BD Biosciences) and SHIV-infected cells were identified by intracellular staining using Alexa fluor 488-conjugated anti-p27 Abs (clone 2F12). Samples were analyzed on an LSRII cytometer (BD Biosciences). Data analysis was performed using FlowJo v10.0.7 (Tree Star). The percentage of ADCC was calculated with the following formula: (% of p27+ cells in Targets plus Effectors) − (% of p27+ cells in Targets plus Effectors plus Abs or sera) / (% of p27+ cells in Targets) by gating on infected living target cells. Of note, samples were deidentified and tested and analyzed blindly.

FACS-based gp120-coated CEM.NKR ADCC assay

CEM.NKR-CCR5-sLTR-Luc cells were coated with 1μg of recombinant HIV-1_{BG505} N332 gp120/million cells for 30 min at 37°C. gp120-coated target cells were used as target cells and were stained with viability (AquaVivid; Thermo Fisher Scientific) and cellular (cell proliferation dye eFluor670; eBioscience) markers. ADCC was performed as described above with the difference that after target/effector cells co-incubation, cells were fixed with a PBS-formaldehyde solution (2% formaldehyde final concentration) containing a constant number of flow cytometry particles ($5x10^4$/ml) (AccuCount Blank Particles, 5.3 μm; Spherotech, Lake Forest, IL, USA). These particles are designed to be used as reference particles since their concentration is known, thus allowing to count the absolute cell number by flow cytometry. A constant number of particles ($1x10^3$) were counted during cytometry acquisition in order to normalize the number of viable targets cells. Each sample was acquired with a LSRII (BD Bioscience, Mississauga, ON, Canada) and data analysis was performed using FlowJo v10.0.7 (Tree Star, Ashland, OR, USA). The percentage of ADCC was calculated with the following formula: (relative count of gp120-coated cells in targets plus effectors) - (relative count of gp120-coated cells in targets plus effectors plus Abs or sera) / (relative count of gp120-coated cells in targets) by gating live target cells (Veillette et al., 2015). Of note, samples were deidentified and tested and analyzed blindly.

T cell analysis

Frozen aliquots of macaque PBMCs were thawed, washed once with RPMI + 10% FBS (R10), incubated with DNase (100ug/ml, StemCell Technologies 07900) for 15 minutes at 37C, then washed again and split in half for a $CD8^+$ ICS assay and a $CD4^+$ T cell Activation Induced Marker (AIM) assay (Reiss et al., 2017).

For the $CD8^+$ T cell ICS assay, the sample was further split into three groups and either left unstimulated (ns), stimulated with BG505 Env peptides (5 μg/ml), or stimulated with SEB (1 μg/ml) for 2 hours at 37°C. Brefeldin A was then added (2 μg/ml), and the stimulations incubated for another 4 hours at 37°C. The cells were then stained for 30 minutes at 4°C with the fluorescent antibodies in the Surface Marker Panel below and washed twice with FACS buffer. They were fixed with eBio intranuclear fix/perm kit for 20 minutes, washed once with perm buffer, then stained with the antibodies in the Intranuclear Panel in perm buffer for 30 minutes at 4°C. The samples were then washed with FACS buffer and acquired on a BD LSR Fortessa.

For the CD4+ T cell AIM assay, the sample was further split into three groups and either left unstimulated (ns) or stimulated with BG505 Env peptides (5 μg/ml), or stimulated with SEB (100 pg/ml) for 24 hours at 37°C. The cells were then stained for 60 minutes at 4°C with the fluorescent antibodies in the AIM Surface Marker Panel below, washed with FACS buffer, fixed with 1% formaldehyde for 10 minutes at 4°C, then washed again before acquisition on a BD LSR Fortessa.

CD8 T cell Surface Marker Panel:

CD4 (Clone SK3) PerCP 1:200
CD20 (Clone 2H7) BV570 1:200
CD8 (Clone RPA-T8) BV650 1:200
CCR5 (3A9) APC 1:200
α4β7 (Act-1) PE 1:200
Live/Dead APC e780 1:1000

CD8 T cell Intranuclear Panel:
Ki67 (B56) Ax488 1:100
IL2 (MQ1-17H12) Ax700 1:100
IFNγ (Clone B27) Pac Blue 1:100
TNF (MAb11) PECy7 1:100
CD40L (24-31) BV605 1:100

CD4 T Cell AIM Surface Marker Panel:
CD4 (Clone OKT-4) BV650 1:100
CD20 (Clone 2H7) BV570 1:100
PD1 (Clone EH12.2H7) BV785 1:100
CXCR5 (Clone MU5UBEE) PECy7 1:100
Live/Dead APC e780 1:1000
CD14 (61D3) APC e780 1:100
CD16 (eBioCD16) APC e780 1:100
CD25 (Clone BC96) FITC 1:100
Ox40 (Clone L106) PE 1:100
4-1-BB (4B4-1) BV421 1:100
ICOS (C398.4A) PerCP Cy5.5 1:100
CXCR3 (1C6) APC 1:100

Full length *env* viral sequencing

Long-read env sequencing

Samples were processed using the full-length Env sequencing protocol developed in (Laird Smith et al., 2016), but with modified primers and PCR conditions. Briefly, plasma samples were pelleted through a sucrose cushion to enrich for virions, RNA was extracted using the QIAamp Viral RNA Mini Kit (part no. 52906; QIAGEN, Valencia, CA), and cDNA generated using the SuperScript III First Strand Synthesis System for RT-PCR (part no. 18080-051; Thermo Fisher, Fremont, CA), with oligo (dT) primers. SHIV *env* was amplified from this cDNA using the HIV *env* forward primer from (Laird Smith et al., 2016) Env-F: GAGCAGAAGACAGTGGCAATGA, and using a reverse primer designed for this SHIV: CCCTGATTGTATTTCTGTCCCTCAC, both purchased (de-salted) from Integrated DNA Technologies (San Diego, CA) and diluted to 20 pmol in 0.1X TE buffer before use. PCR was as in (Laird Smith et al., 2016), using the Advantage 2 PCR reaction mixture (Advantage 2 PCR Kit, catalog no. 639206; Clontech, Mountain View, CA), with the SA Buffer, but using 42 cycles of 15 s denaturation at 95°C, 30 s annealing at 64°C, and 3 min extension at 68°C. A QIAquick PCR Purification Kit (part no. 28106; QIAGEN, Valencia, CA) was used to purify PCR products, and Pacific Biosciences library preparation was exactly as in (Laird Smith et al., 2016), but using the newer P6/C4 chemistry, and with a modified 0.025nM loading concentration, and a 6 hour movie time. The challenge stock was handled identically but was highly concentrated and thus only 23 PCR cycles were used during amplification.

PacBio env data processing

An updated version of the Full-Length Env Analyzer (FLEA) (Eren et al., 2017; Laird Smith et al., 2016) pipeline was used to process SIV PacBio reads. Briefly, PacBio's CCS2 algorithm was used to reconstruct single molecule Circular Consensus Sequence (CCS) reads, outputting fastq files. These reads were filtered for length, quality, and for matching an Env reference database (here we included the known BG505.SHIV challenge sequence) with FLEA's default parameter settings. FLEA's error correction and data-summarizing approach was used, again with default parameters, collapsing near-identical reads and generating high-quality consensus sequences (HQCSs), along with HQCS frequencies, which are then codon aligned. These HQCS sequences are visualized in a web browser environment, allowing the exploration of immunotype frequencies, and displaying variants upon the leaf nodes of a maximum likelihood phylogeny. Variant frequencies in Figures 4A–4B were computed from HQCS sequence frequencies.

Complex preparation for negative-stain EM

Serum Fab preparation was carried out as previously described (Bianchi et al., 2018). In brief, after buffer exchanging into TBS, up to ~1 mg of total Fab was incubated overnight with 10-15 μg BG505 trimers at RT in ~50 μL total volume. Complexes were then purified via size exclusion chromatography (SEC) using Superose 6 Increase 10/300 column (GE Healthcare) in order to remove unbound Fab. The flow-through fractions containing the complexes were pooled and concentrated using 100 kDa cutoff centrifugal filters (EMD Millipore). The final trimer concentration was titrated to 0.04 mg/mL prior to application onto carbon-coated copper grids.

Negative-stain EM

The SEC-purified complexes were applied to glow-discharged, carbon-coated 400-mesh copper grids, followed by pipetting 3 μl of 2% (w/v) uranyl formate stain and blotting, followed by application of another 3 μl of stain for 45–60 s, again followed by blotting. Stained grids were stored under ambient conditions until ready for imaging. Images were collected via Leginon software using a Tecnai T12 electron microscopes operated at 120 kV and 52,000x magnification. In all cases, the electron dose was 25 $e^-/Å^2$. Particles were picked from the raw images using DoG Picker and placed into stacks using Appion software. 2D reference-free alignment was performed using iterative MSA/MRA. The particle stacks were then converted from IMAGIC to RELION-formatted MRC stacks and subjected to RELION 2.1 2D and 3D classification. A detailed protocol can be found in Bianchi et al., Immunity 2018.

QUANTIFICATION AND STATISTICAL ANALYSIS

Infection probability per challenge event was modeled as depending on the BG505 N332 S375Y log10 ID_{50} nAb titer at the time of challenge using a modified logistic regression, where the maximum infection probability (where $0 < max < 1$) was an additional parameter to be estimated by the model, rather than being fixed at 1 as in traditional logistic regression:

$$\frac{max}{1+e^{-slope\ (x-offset)}}$$

This adjustment is necessary because unimmunized animals with no serum nAb titers are not infected with 100% probability upon the first challenge, as a consequence of the chosen AID_{75} challenge dose. The infection event was assumed to be the challenge time point prior to the detection of viremia. Per-time point challenge outcomes were assumed to be conditionally independent of each other when conditioning on the corresponding BG505 N332 S375Y log10 ID_{50} nAb titer of the respective time point. We assumed weakly informative priors over the three model parameters, with slope~*Normal*(0,10), offset~*Normal*(0,10), and max~*Uniform*(0,1), and we used the Metropolis algorithm to draw 1 million samples from the posterior distribution. Chain mixing was rapid (see trace plots in Figure S5B), with effective sample sizes (ESSs) above 20,000 for all 3 parameters and for the log posterior probability. The posterior parameter distributions are visualized in Figure S5A. The calculated 5%, 50%, and 95% quantiles for each parameter were:

slope: −6.32937, −3.49356, −1.99455
offset: 1.71374, 2.12005, 2.4339
max: 0.602868, 0.80477, 0.962382

While under the prior distribution, P(slope < 0) = 0.5 and P(slope > 0) = 0.5, allowing equal prior probability of protective or sensitizing effects of neutralizing antibodies, the posterior probability of P(slope < 0) = 1 indicated the strongest possible evidence for decreasing infection probabilities given increasing ID_{50} nAb titers. Figure S5C shows 10,000 posterior sampled logistic curves, and the 5%, median, and 95% credible intervals for the infection probability computed from these, that were used to plot Figure 5C.

Graphpad Prism v7.0 was used for all standard statistical analyses. The significance of differences in neutralization and binding data between groups was calculated using unpaired, two-tailed Mann-Whitney U tests, correlations were calculated using Spearman correlation tests. Statistical parameters of all analyses are reported in the respective figure legends.

Immunity, Volume *50*

Supplemental Information

Vaccine-Induced Protection from Homologous Tier 2 SHIV Challenge in Nonhuman Primates Depends on Serum-Neutralizing Antibody Titers

Matthias G. Pauthner, Joseph P. Nkolola, Colin Havenar-Daughton, Ben Murrell, Samantha M. Reiss, Raiza Bastidas, Jérémie Prévost, Rebecca Nedellec, Benjamin von Bredow, Peter Abbink, Christopher A. Cottrell, Daniel W. Kulp, Talar Tokatlian, Bartek Nogal, Matteo Bianchi, Hui Li, Jeong Hyun Lee, Salvatore T. Butera, David T. Evans, Lars Hangartner, Andrés Finzi, Ian A. Wilson, Richard T. Wyatt, Darrell J. Irvine, William R. Schief, Andrew B. Ward, Rogier W. Sanders, Shane Crotty, George M. Shaw, Dan H. Barouch, and Dennis R. Burton

SUPPLEMENTARY MATERIALS

Figure S1: Selection of challenge animals from previously immunized macaques.
Figure S2: Viral load of unimmunized animals after viral challenge.
Figure S3: NAb titers from protected animals remain stable over one year past immunization
Figure S4: Neutralizing antibody titers 7 days before the detection of viremia.
Figure S5: Bayesian logistical regression of serum nAb titer and infection probablility.
Figure S6: Additional analyses of $CD4^+$ and $CD8^+$ T cell activation at week 0.
Figure S7: Additional analysis of ADCC activity from control mAbs and animal sera at week 0.

Table S1: Genotyping of macaques used in the immunization study.
Table S2: Estimated frequency of MOI in macaques following SHIV challenge.
Table S3: Survival models and statistical end point analyses.

A

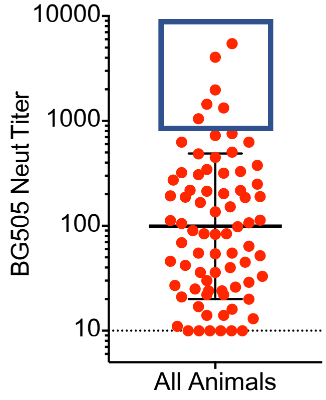

B

Immunogen	Animal ID	Peak BG505 nAb-titer		Animal ID	Immunogen
BG505 SOSIP v4.1	12-137	1979	54	12-060	BG505 SOSIP v4.1
BG505 SOSIP v4.1	12-046	1328	90	12-065	BG505 SOSIP v5.2
BG505 Olio6	12-143	1048	40	12-149	BG505 Olio6 CD4-KO
BG505 SOSIP v5.2 (Pump)	12M169	4047	25	12-153	BG505 SOSIP v5.2
BG505 SOSIP v5.2 (Pump)	11M088	1440	64	12M248	BG505 SOSIP v5.2 (Pump)
BG505 Olio6 CD4-KO (Lipo)	4O9	5453	13	0Q7	BG505 SOSIP.664 His (Lipo)

C

	Animal ID	Peak Titer nAb-titer	Gender	Age [y]	Weight [kg]
High nAb-Titer Group	12-137	1979	Female	4.3	4.1
	12-046	1328	Male	4.5	5.3
	12-143	1048	Female	4.1	3.3
	12M169	4047	Female	4.4	4.2
	11M088	1440	Female	5.5	7.4
	4O9	5453	Female	4.0	4.5
Low nAb-Titer Group	12-060	54	Male	4.4	5.7
	12-065	90	Female	4.5	4.5
	12-149	40	Female	4.0	3.6
	12-153	25	Female	4.0	3.8
	12M248	64	Female	4.4	4.7
	0Q7	13	Female	4.1	5.0
			**Average**	**4.4**	**4.7**
			**SD**	**0.4**	**1.1**

Figure S1, related to Figure 1. Selection of challenge animals from previously immunized macaques. (A-C) Challenge animals were selected from 78 macaques previously immunized with the indicated native-like BG505 trimer proteins (de Taeye et al., 2015; Kulp et al., 2017; Sanders et al., 2013; Torrents de la Peña et al., 2017) at weeks 0, 8 and 24 (Pauthner et al., 2017). Immunogens used in the study differ only in the precise details of the modifications made to stabilize the V3 loop; detailed accounts of differences can be found in the respective publications cited above. Some trimers additionally included a mutation to reduce binding to human CD4 (CD4-KO), which

minimally affects rhesus CD4 binding. All bolus-immunized animals received 100µg adjuvanted trimer per immunization; osmotic pump-immunized animals (Pump) received 200µg adjuvanted trimer per immunization. (A) 6 macaques with the highest peak autologous BG505 neutralization titers at week 26 were selected for the high nAb titer group in this study as shown in the blue box. (**B**) High nAb titer animals were carefully matched with animals that received identical or very similar immunization regimens but had developed low peak (week 26) autologous nAb titers. (**C**) High- and low-titer animals were closely matched for gender, age and weight.

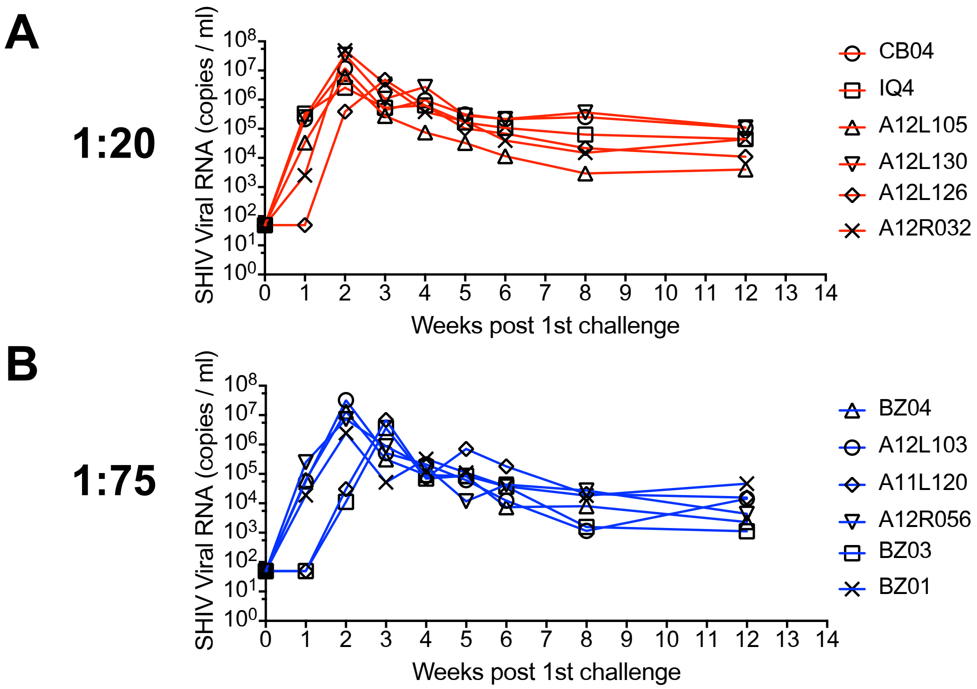

Figure S2, related to Figure 2. Viral load of unimmunized animals after viral challenge. Viral load curves of the 1:20 (1 x 10^8 RNA copies/ml) (**A**) and 1: 75 (2.8 x 10^7 RNA copies/ml) titration control group animals (**B**). 2.8 x 10^7 viral RNA copies/ml, or 1.4 x 10^7 virions, infected at least 4/6 animals with the 1st challenge and the remaining 2 animals following the 2nd challenge. The 1:75 challenge dose was subsequently chosen for the main study.

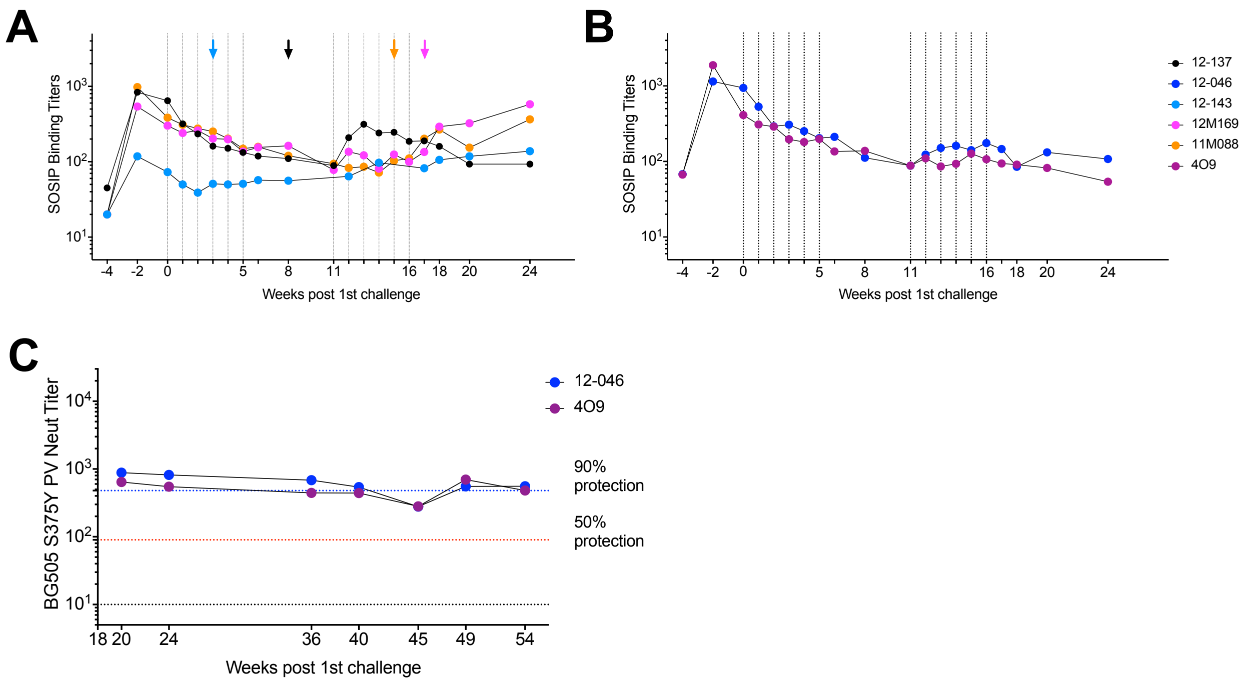

Figure S3, related to Figure 3. NAb titers from protected animals remain stable over one year past immunization (**A-B**) ELISA binding titers in fully protected and infected animals. BG505 SOSIP.664 ELISA EC_{50} binding titers in the high nAb titer group at indicated time points for animals that became infected over the course of 12 challenges (**A**) and fully protected animals (**B**). First detection of plasma viremia is indicated by colored arrows corresponding to the animal IDs shown in the figure legend. (**C**) Longitudinal BG505 S375Y pseudovirus ID_{50} nAb titers in fully protected animals. ID_{50} nAb titers in protected animals remain relatively stable after an initial decline for over 1 year past the final immunization. 90% and 50% protective nAb titer levels as determined in Figure 5C are shown as dotted blue and red lines, respectively.

A

High Titer Group	nAb titer ID50 (ID80) 7d before infection detected	nAb titer ID50 (ID80) at week 20 still uninfected
12-137*	494 (123)	
12-046		958 (239)
12-143	458 (115)	
12M169	141 (35)	
11M088	248 (62)	
4O9		754 (189)
**Geo Mean**	**299 (75)**	**850 (212)**

*14d before infection was detected

Low Titer Group	nAb titer ID50 (ID80) 7d before infection detected	nAb titer ID50 (ID80) at week 20 still uninfected
12-060	110 (27)	
12-153	174 (43)	
12-065	202 (51)	
12-149	57 (< 20)	
12M248	136 (34)	
0Q7	20 (< 20)	
**Geo Mean**	**92 (21)**	

B

High Titer Group	nAb titer ID50 (ID80) 7d before infection detected	nAb titer ID50 (ID80) at week 20 still uninfected
12-137*	26 (< 20)	
12-046		52 (< 20)
12-143	39 (< 20)	
12M169	< 20 (< 20)	
11M088	23 (< 20)	
4O9		40 (< 20)
	**26 (<20)**	**46 (<20)**

*14d before infection was detected

Low Titer Group	nAb titer ID50 (ID80) 7d before infection detected	nAb titer ID50 (ID80) at week 20 still uninfected
12-060	< 20 (< 20)	
12-065	< 20 (< 20)	
12-149	< 20 (< 20)	
12-153	< 20 (< 20)	
12M248	< 20 (< 20)	
0Q7	< 20 (< 20)	
	**< 20** (< 20)	

C

	TZM-bl Neutralization					
	**BG505 Pseudovirus**	**BG505 S375Y Pseudovirus**	**SHIV$_{BG505}$ S375Y Pseudovirus**	**SHIV$_{BG505}$ S375Y IMC (293T-grown)**	**SHIV$_{BG505}$ S375Y rh-CD4 challenge stock**	**ΔSHIV$_{BG505}$ rh-CD4 / 293T**
PGT121	0.01	0.01	0.01	0.01	0.04	3
PGT128	0.004	0.01	0.005	0.01	0.03	3
PGT135	> 5	> 5	> 5	> 5	> 5	
PGT145	0.01	0.003	0.08	0.01	0.06	6
CAP08	0.005	0.003	0.002	0.01	0.005	1
CH03	0.19	0.21	0.34	0.20	1.89	9
VRC01	0.09	0.12	0.19	0.13	0.88	7
b12	> 5	> 5	> 5	> 5	> 5	
PGT151	0.001	0.001	0.001	0.002	0.004	2
4E10	0.99	0.89	0.74	1.54	> 5	<3
10E8	0.07	0.21	0.25	0.14	1.58	12

Figure S4, related to Figure 5. Neutralizing antibody titers 7 days before the detection of viremia. (**A-B**) ID_{50} and ID_{80} neutralization titers of high and low nAb titer animals against BG505 N332 S375Y pseudovirus (**A**), as well as rhCD4+ T-cell-grown $SHIV_{BG505}$ N332 S375Y challenge stock (**B**), 7 days before the detection of viremia in the blood. Titers are averaged from three independent experiments. All assays used TZM-bl target cells. (**C**) Comparison of bnAb IC_{50} neutralization titers (µg/ml) against BG505 N332 pseudovirus, BG505 N332 S375Y pseudovirus, $SHIV_{BG505}$ N332 S375Y pseudovirus and $SHIV_{BG505}$ N332 S375Y infectious molecular clone (IMC) grown in HEK-293T cells (293T-grown), as well as rhCD4+ T-cell-grown (rh-CD4) $SHIV_{BG505}$ N332

S375Y challenge stock. The ratio between the latter two is shown on the right. BG505 N332 S375Y pseudovirus showed similar IC_{50} values as $SHIV_{BG505}$ N332 S375Y IMC grown in HEK-293T cells. Deep sequencing analysis of the challenge stock (see also Figure 4A-B) showed no consensus mutations between the challenge stock and pseudovirus sequence *env* sequences. Conversely, the challenge stock is neutralized to ~100% completion by potent animal sera, indicating that no sizable resistant viral populations exist in the challenge stock. Uncolored ratios between rhCD4+ T-cell and HEK-293T cell-grown $SHIV_{BG505}$ are shown in the last column.

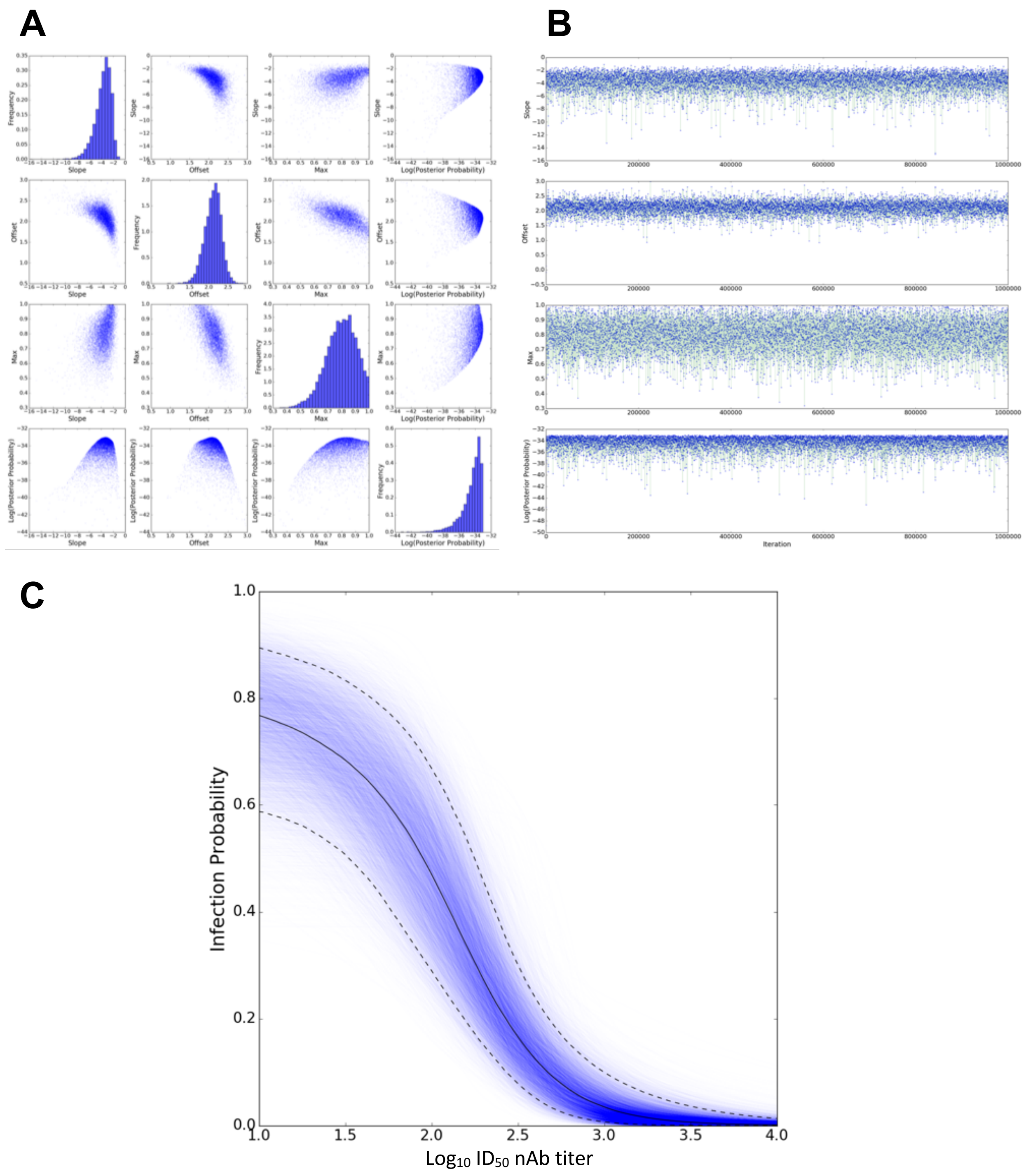

Figure S5, related to Figure 5. Bayesian logistical regression of serum nAb titer and infection probablility. (A) Pairwise scatter plots and histograms of 10,000 samples from the posterior distribution of our modified Bayesian logistic regression model. (**B**) Markov Chain Monte Carlo (MCMC) trace plots for the slope, offset and max parameters of our modified Bayesian logistic regression model, showing rapid mixing and high effective sample sizes (ESSs) above 20,000, indicating strong chain convergence and reliable

posterior inference. (**C**) 10,000 logistic curves were drawn from the posterior distribution of our Bayesian infection probability model (thin blue lines), and 5%, median, and 95% credible intervals computed from these posterior samples. This graph depicts the full extent of the model's uncertainty in the infection probability given the logarithmic ID_{50} BG505 N332 S375Y pseudovirus nAb titer.

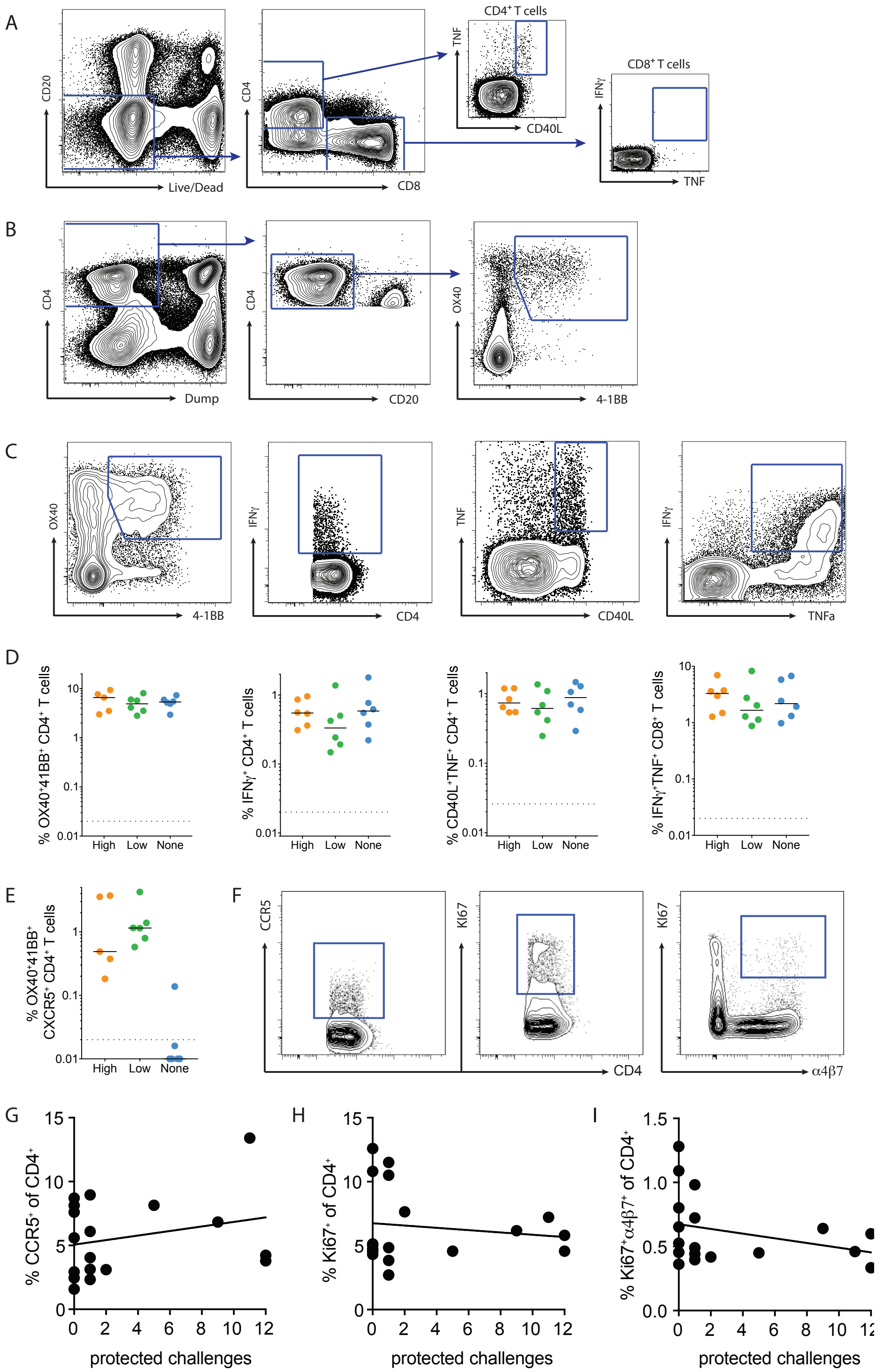

Figure S6, related to Figure 6. Additional analyses of CD4⁺ and CD8⁺ T cell activation at week 0 (day of the first challenge). (**A**) Gating strategy for ICS cytokine

expression by $CD4^+$ and $CD8^+$ T cells. (**B**) Gating strategy for detecting Env-specific $CD4^+$ T cells via the OX40/4-1BB AIM assay. Dump includes Live/Dead Viability Dye, CD14, and CD16. (**C**) Representative flow plots of the SEB-stimulated positive control for the $CD4^+$ T cell AIM assay, the $CD4^+$ T cell ICS assays, and the $CD8^+$ T cell ICS assay. (**D**) Quantification of the SEB response for each of the assays in (**C**). Signal from the unstimulated condition was subtracted from the SEB signal for each sample. (**E**) Quantification of the percent of $CXCR5^+$ $CD4^+$ T cells that express OX40 and 4-1BB following antigen stimulation in the $CD4^+$ T cell assay (unstimulated condition subtracted). (**F**) Representative flow plots of CCR5, Ki67, and α4β7 expression on $CD4^+$ T cells from PBMC. (**G-I**) Correlations between the percent of $CD4^+$ T cells that express CCR5 (**G**), Ki67 (**H**), or Ki67 and α4β7 (**I**) with the number of challenges against which the animal was successfully protected.

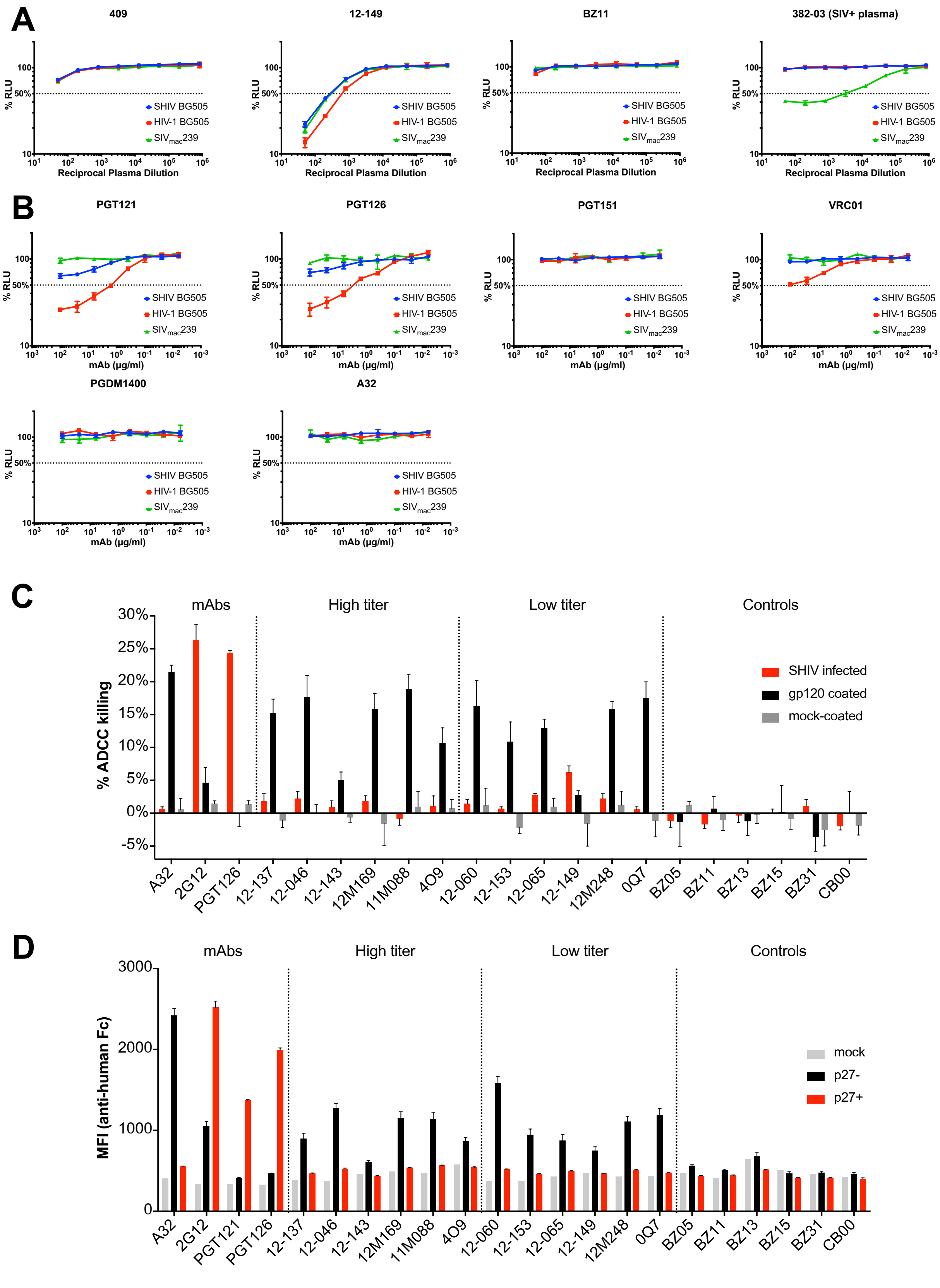
A
409
12-149
BZ11
382-03 (SIV+ plasma)
% RLU
Reciprocal Plasma Dilution
SHIV BG505
HIV-1 BG505
SIVmac239
B
PGT121
PGT126
PGT151
VRC01
PGDM1400
A32
mAb (µg/ml)
C
mAbs
High titer
Low titer
Controls
% ADCC killing
SHIV infected
gp120 coated
mock-coated
A32
2G12
PGT126
12-137
12-046
12-143
12M169
11M088
4O9
12-060
12-153
12-065
12-149
12M248
0Q7
BZ05
BZ11
BZ13
BZ15
BZ31
CB00
D
MFI (anti-human Fc)
mock
p27-
p27+
PGT121

Figure S7, related to Figure 7. Additional analysis of ADCC activity from control mAbs and animal sera at week 0 (day of the first challenge). (**A**) To control for strain specificity of ADCC activity, all immunized animals were tested against $SHIV_{BG505}$ S375Y, HIV-1 BG505 and SIV_{mac}239-infected CEM cells. Shown is the serum ADCC activity from a representative high nAb titer group animal (4O9), the only immunized animal that showed nonspecific ADCC activity (12-149), a representative unimmunized animal (BZ11) as well as serum from a SIV_{mac}239 infected control animal from an unrelated study (382-03). **(B)** As additional positive and negative controls for ADCC activity, a panel of bnAbs and A32, a cluster A specific non-neutralizing antibody, were evaluated as shown. BnAbs showed differing abilities to mediate ADCC against SHIV and SIV-infected cells but showed no activity against SIVmac239-infected cells. The nnAb A32 did not mediate ADCC against HIV or SHIV-infected cells (Bredow et al., 2016; Ding et al., 2016)**,** in line with its inability to bind intact Env trimers. (**C-D**) ADCC measurement and Env-staining of gp120-coated or SHIV infected CEM.NKR cells using mAbs and macaque sera from week 0 (day of the first challenge). (**C**) Comparison of flow cytometry-assessed ADCC killing of CEM.NKR target cells that were either infected with the $SHIV_{BG505}$ S375Y challenge stock (SHIV infected), coated with BG505 gp120 (gp120 coated) or uninfected/uncoated (mock-coated). (**D**) Mean fluorescent intensity (MFI) of $SHIV_{BG505}$ S375Y challenge stock infected or uninfected (mock) CEM.NKR target cells stained with indicated mAbs and sera. $SHIV_{BG505}$ S375Y exposed target cells were gated for productively infected cells (p27+) and uninfected cells (p27-). While p27+ target cells showed little specific staining by animal sera, p27- cells showed various levels of serum staining, indicating gp120 coating of uninfected bystander cells by shed gp120 molecules from productively infected cells. Strong binding by CD4-induced specific mAb A32 to p27-, but not p27+ cells, confirms that uninfected bystander T cells bind shed gp120 via CD4 and thus present gp120 in the CD4-bound state, which is only weakly bound by PGT126. All assays used animal sera diluted 1:250 or mAbs at 5µg/ml. All panels show an average of three independent experiments.

Table S1, related to Figure 1: Genotyping of macaques used in the immunization study. All animals were genotyped for known Mamu (A*01, B*08, B*17) and TRIM-5α alleles (TFP, Q) associated with host restriction of certain SIV strains in nonhuman primates. However, $SHIV_{BG505}$ S375Y uses the SIVmac251-derived SIVmac766 sequence as its genomic backbone to enable replication in macaques (Li et al., 2016). SIVmac251 was shown to be unaffected by otherwise restrictive or partially restrictive TRIM-5α genotypes (TFP/TFP, TFP/Q) *in vivo* (Fenizia et al., 2011). Moreover, SIVmac766 has the identical N-terminal p27 capsid sequence as SIVmac239 (87-APQQGQLREPSGSDIAGTT-SSVDEQIQW-113), which was shown to confer resistance to TRIM-mediated restriction in cell culture experiments (Kirmaier et al., 2010). Consistent with these reports, we observed no delayed onset or reduction of peak viremia in TRIM-5$\alpha^{TFP/TFP}$ homozygous control animals (see for example Figure S2).

Group	Animal ID	Gender	DOB	A*01	B*08	B*17	TRIM	Alleles
High Titer Group	12-137	Female	6/16/12	(-)	(-)	(-)	1-5/6-11	TFP/Q
	12-046	Male	4/1/12	(-)	(-)	(-)	6-11/6-11	Q/Q
	12-143	Female	8/28/12	(-)	(-)	(-)	6-11/6-11	Q/Q
	12M169	Female	5/5/12	(-)	(-)	(-)	1-5/6-11	TFP/Q
	11M088	Female	4/23/11	(-)	(-)	(-)	1-5/6-11	TFP/Q
	4O9	Female	10/1/12	(-)	(-)	(-)	1-5/6-11	TFP/Q
Low Titer Group	12-060	Male	5/26/12	(-)	(-)	(+)	1-5/6-11	TFP/Q
	12-153	Female	10/4/12	(-)	(-)	(-)	1-5/6-11	TFP/Q
	12-065	Female	4/15/12	(-)	(-)	(+)	1-5/6-11	TFP/Q
	12-149	Female	10/1/12	(+)	(-)	(-)	1-5/6-11	TFP/Q
	12M248	Female	6/7/12	(-)	(-)	(-)	1-5/6-11	TFP/Q
	0Q7	Female	9/14/12	(-)	(+)	(-)	6-11/6-11	Q/Q
Unimmunized (Concurrent)	BZ05	Male	6/10/12	(-)	(-)	(-)	1-5/1-5	TFP/TFP
	BZ11	Male	6/11/12	(+)	(-)	(-)	1-5/6-11	TFP/Q
	BZ13	Female	3/21/12	(+)	(-)	(-)	1-5/6-11	TFP/Q
	BZ14	Female	6/4/12	(-)	(-)	(-)	1-5/6-11	TFP/Q
	BZ15	Male	6/18/12	(-)	(-)	(-)	1-5/6-11	TFP/Q
	BZ31	Male	4/28/12	(-)	(-)	(-)	1-5/6-11	TFP/Q
	CB00	Male	4/12/12	(-)	(-)	(-)	6-11/6-11	Q/Q
Unimmunized (1:75 Titration)	BZ01	Female	5/15/12	(+)	(-)	(-)	6-11/6-11	Q/Q
	BZ03	Female	5/19/12	(+)	(-)	(-)	1-5/6-11	TFP/Q
	BZ04	Male	3/26/12	(-)	(+)	(-)	1-5/1-5	TFP/TFP
	A12R056	Male	7/1/12	(-)	(-)	(-)	1-5/1-5	TFP/TFP
	A11L120	Male	6/20/12	(-)	(-)	(-)	1-5/6-11	TFP/Q
	A12L103	Male	6/12/12	(-)	(+)	(-)	1-5/1-5	TFP/TFP
Unimmunized (1:20 Titration)	CB04	Male	7/25/12	(-)	(-)	(-)	1-5/1-5	TFP/TFP
	IQ4	Female	9/9/12	(-)	(-)	(-)	1-5/1-5	TFP/TFP
	A12L105	Female	6/14/12	(+)	(-)	(-)	6-11/6-11	Q/Q
	A12L130	Male	7/21/12	(-)	(-)	(-)	1-5/6-11	TFP/Q
	A12L126	Male	7/11/12	(-)	(-)	(-)	1-5/6-11	TFP/Q
	A12R032	Male	6/3/12	(-)	(-)	(-)	1-5/6-11	TFP/Q

Table S2, related to Figure 2: Estimated frequency of MOI in macaques following SHIV challenge. Estimated frequency (number per 100 animals) of indicated multiplicities of infection (MOIs) as a function of the chosen animal infectious dose (AID) for all challenged animals (upper table) or infected animals only (lower table). MOI frequencies are calculated using the following function: f(MOI) = 100 * dpois(MOI, -ln(1-AID/100)), where dpois is the probability density function for the Poisson distribution, AID is the AID number, e.g. 50 for an AID_{50}, and ln is the natural logarithm function. For an AID_{75} that was used in this study, following a single challenge, 35% of animals are estimated to be productively infected with 1 virion (MOI = 1), 24% with 2 virions (MOI = 2) and 16% with 3 or more virions (MOI > 2). Thus, among infected animals, 35 of 75 animals, or 46%, are expected to be productively infected with 1 virion, 32% with 2 virions, and 22% with 3 or more virions. The observed MOI-distributions for HIV-1 infection in human cohorts do not strictly follow a Poisson distribution of infrequent independent events, due to heterogeneity in MOI-affecting transmission risk-factors (e.g. integrity of genital mucosae) that cannot be controlled for in human cohorts (Abrahams et al., 2009). However, it is reasonable to apply the above method to estimate MOI-distributions in the setting of controlled atraumatic intrarectal challenge of macaques with SHIVs, where observed MOI-distributions do approximate a Poisson distribution (Liu et al., 2010).

Estimated Frequency of Multiplicity of Infection (MOI) - Total Animals

	**0**	**1**	**2**	**3**	**4**	**5**	**6**	**7**	**8**	**9**	**10**
AID_{99}	1	5	11	16	19	17	13	9	5	3	1
AID_{90}	10	23	27	20	12	5	2	1	0	0	0
AID_{75}	25	35	24	11	4	1	0	0	0	0	0
AID_{50}	50	35	12	3	0	0	0	0	0	0	0
AID_{25}	75	22	3	0	0	0	0	0	0	0	0
AID_{10}	90	9	0	0	0	0	0	0	0	0	0

Estimated Frequency of Multiplicity of Infection (MOI) - Infected Animals

	**1**	**2**	**3**	**4**	**5**	**6**	**7**	**8**	**9**	**10**
AID_{99}	5	11	16	19	17	13	9	5	3	1
AID_{90}	26	29	23	13	6	2	1	0	0	0
AID_{75}	46	32	15	5	1	0	0	0	0	0
AID_{50}	69	24	6	1	0	0	0	0	0	0
AID_{25}	86	12	1	0	0	0	0	0	0	0
AID_{10}	95	5	0	0	0	0	0	0	0	0

Table S3, related to Figure 2. Survival models and statistical end point analyses. Statistical analysis of survival model and end point parameters for all animals after 6 consecutive challenges at week 8 (upper table) or following all 12 challenges, including a 5-week resting period between challenge sets, at week 20 (lower table). All indicated tests were calculated in Graphpad PRISM v7.0a.

	Survival Model (Kaplan-Meier)			End point contingency analysis		
	P-Value vs Controls[1]	P-Value vs Controls[2]	Median Protection	Complete Protection	P-Value vs Control[3]	P-Value vs Control[4]
High Titer (N=6)	0.0002 ***	<0.0001 ****	-	67%	0.0013 **	0.0049 **
Low Titer (N=6)	0.0967	0.0967	2 challenges	0%	-	>0.99
Controls (N=12)	N/A	N/A	1 challange	0%	N/A	N/A
	[1]Gehan-Breslow-Wilcoxon test; [2]Log-rank (Mantel-Cox) test			[3]Chi-square test; [4]Fisher's Exact test		

	Survival Model (Kaplan-Meier)			End point contingency analysis		
	P-Value vs Controls[1]	P-Value vs Controls[2]	Median Protection	Complete Protection	P-Value vs Control[3]	P-Value vs Control[4]
High Titer (N=6)	0.0002 ***	<0.0001 ****	11 challenges	33%	0.0339 *	0.098
Low Titer (N=6)	0.0967	0.0967	2 challenges	0%	-	>0.99
Controls (N=12)	N/A	N/A	1 challange	0%	N/A	N/A
	[1]Gehan-Breslow-Wilcoxon test; [2]Log-rank (Mantel-Cox) test			[3]Chi-square test; [4]Fisher's Exact test		
